# X chromosome-wide association study of quantitative biomarkers from the Alzheimer’s Disease Neuroimaging Initiative study

**DOI:** 10.3389/fnagi.2023.1277731

**Published:** 2023-11-14

**Authors:** Kai-Wen Wang, Yu-Xin Yuan, Bin Zhu, Yi Zhang, Yi-Fang Wei, Fan-Shuo Meng, Shun Zhang, Jing-Xuan Wang, Ji-Yuan Zhou, for the Alzheimer’s Disease Neuroimaging Initiative

**Affiliations:** ^1^State Key Laboratory of Organ Failure Research, Ministry of Education, Guangdong Provincial Key Laboratory of Tropical Disease Research, Department of Biostatistics, School of Public Health, Southern Medical University, Guangzhou, China; ^2^Guangdong-Hong Kong-Macao Joint Laboratory for Contaminants Exposure and Health, Guangzhou, China

**Keywords:** Alzheimer’s disease, X chromosome-wide association study, quantitative biomarker, cross-sectional study, longitudinal study

## Abstract

**Introduction:**

Alzheimer’s disease (AD) is a complex neurodegenerative disease with high heritability. Compared to autosomes, a higher proportion of disorder-associated genes on X chromosome are expressed in the brain. However, only a few studies focused on the identification of the susceptibility loci for AD on X chromosome.

**Methods:**

Using the data from the Alzheimer’s Disease Neuroimaging Initiative Study, we conducted an X chromosome-wide association study between 16 AD quantitative biomarkers and 19,692 single nucleotide polymorphisms (SNPs) based on both the cross-sectional and longitudinal studies.

**Results:**

We identified 15 SNPs statistically significantly associated with different quantitative biomarkers of the AD. For the cross-sectional study, six SNPs (rs5927116, rs4596772, rs5929538, rs2213488, rs5920524, and rs5945306) are located in or near to six genes *DMD*, *TBX22*, *LOC101928437*, *TENM1*, *SPANXN1*, and *ZFP92*, which have been reported to be associated with schizophrenia or neuropsychiatric diseases in literature. For the longitudinal study, four SNPs (rs4829868, rs5931111, rs6540385, and rs763320) are included in or near to two genes *RAC1P4* and *AFF2*, which have been demonstrated to be associated with brain development or intellectual disability in literature, while the functional annotations of other five novel SNPs (rs12157031, rs428303, rs5953487, rs10284107, and rs5955016) have not been found.

**Discussion:**

15 SNPs were found statistically significantly associated with the quantitative biomarkers of the AD. Follow-up study in molecular genetics is needed to verify whether they are indeed related to AD. The findings in this article expand our understanding of the role of the X chromosome in exploring disease susceptibility, introduce new insights into the molecular genetics behind the AD, and may provide a mechanistic clue to further AD-related studies.

## 1. Introduction

Alzheimer’s disease (AD) is one of the most common neurodegenerative disorders worldwide which progressively destroys brain function (Lee et al., 2017; De Velasco Oriol et al., 2019; Li et al., 2022). As a type of dementia, AD causes memory loss and cognitive impairment with deficits in executive, language and/or visuospatial functions (Nikolac Perkovic et al., 2021). So far, more than 50 million people around the world have lived with dementia, and approximately 60–80% of them have suffered from the AD (Wang et al., 2021; Fareed et al., 2022), which imposes social, psychological and economic burdens on patients (Andrews et al., 2020). It has been reported that genetic factors play an important role in developing the AD, with an estimated heritability being between 58 and 74% (Roussotte et al., 2014). Thus, it is necessary to further explore genetic determinants of the AD through genome-wide association studies (GWAS) for the disease modeling.

Currently, there have been many GWAS for the AD focusing on qualitative traits (case–control design) (Lambert et al., 2013; Jansen et al., 2019; Kunkle et al., 2019; Yuan et al., 2019). For example, a large case–control study including more than 74,000 subjects found over 21 loci associated with the AD, yet the contribution of each locus to genetic variation is small (Del-Aguila et al., 2018). It is worth noting that, there are several advantages in studying quantitative traits compared to qualitative traits, including higher statistical power and more objective interpretation of results (Yuan et al., 2019). Therefore, some key quantitative biomarkers (QBs) of the AD have been used in GWAS. Specifically, Kim et al. (2011) performed a study of cerebrospinal fluid (CSF) biomarkers and found that single nucleotide polymorphisms (SNPs) rs429358, rs2075650, rs439401, and rs4499362, respectively located in the *APOE*, *TOMM40*, *LOC100129500*, and *EPC2* gene regions, are statistically significantly associated with one or more CSF biomarkers. Among them, *APOE*, *TOMM40*, and *LOC100129500* are known to be important genetic risk factors for the AD. More specifically, there are three most common alleles ε2, ε3, and ε4 in the *APOE* gene, which correspond to three protein isoforms: *APOE2*, *APOE3*, and *APOE4*, respectively (Safieh et al., 2019; Zhou et al., 2023). Genetically, the ε4 allele of the *APOE* gene is the strongest risk factor for the AD (Liu et al., 2013). Evidence suggests that heterozygous carriers of an ε4 allele are 3–4 times more likely to develop the AD than noncarriers (Colovati et al., 2020), so GWAS for the AD typically include *APOE4* allelic dosage (i.e., the number of ε4 alleles in a subject’s *APOE* genotype) as a covariate (Mormino et al., 2016; Wang et al., 2021; Schneider et al., 2022). The *EPC2* gene belonging to the polycomb protein family is involved in heterochromatin formation, and chromatin remodeling may play a role in neurodegenerative diseases such as AD (Kim et al., 2011). Potkin et al. (2009) used hippocampal atrophy measured on magnetic resonance imaging (MRI) as an objectively defined QB, and identified genes *PRUNE2, MAGI2, ARSB, EFNA5*, and *CAND1*, which may be related to the regulation of neuron loss and neural development in the hippocampus. On the other hand, the availability of the data from the Alzheimer’s Disease Neuroimaging Initiative (ADNI) has facilitated a range of analyses on QBs of cognitive, imaging and other biomarkers, demonstrating the strength of multimodal quantitative phenotypic data to identify novel genetic variants (Shen et al., 2014). To date, previous studies on the ADNI database have used the cognitive biomarkers (Hu et al., 2011; Keenan et al., 2012), the CSF biomarkers (Han et al., 2010; Kim et al., 2011; Cruchaga et al., 2013), or the neuroimaging biomarkers (Potkin et al., 2009; Stein et al., 2010; Furney et al., 2011). However, not so many studies explicitly analyzed multiple types of QBs across subjects within the same cohort (Lee et al., 2022). Hence, it is necessary to conduct GWAS for different types of QBs from the ADNI database.

However, most previous analyses about QBs of the AD only considered cross-sectional studies at a certain visit, not taking account of temporal features (Shi et al., 2021). In fact, the pathological progression of the AD is a longitudinal process. So, several researches (Whitwell et al., 2007; Yendiki et al., 2016; Kleineidam, 2020) have conducted longitudinal studies of the AD to better understand cognitive development and disease progression (Huang et al., 2017). For instance, Lim et al. (2022) analyzed some longitudinal cognitive biomarkers to detect cognitive changes in the development of the AD. They found that the subjects with mild cognitive impairment (MCI) and mild AD showed significant declines in verbal episodic memory performance compared to the cognitively normal subjects during their follow-up period of more than 18 months. Notably, the ADNI collected a rich set of longitudinal data, which allowed us to observe the longitudinal trajectory of specific QB. A case in point is that, Lee et al. (2022) conducted an analysis of key QBs from the ADNI database and identified a novel SNP rs5011804 at 12p12.1, which is significantly associated with three cognitive traits and one imaging trait. But this article only conducted a cross-sectional study at each visit separately, not a longitudinal study for their analysis. On the other hand, Ramanan et al. (2015) used the ADNI database to carry out the first GWAS of amyloid accumulation in a longitudinal framework, and identified the gene *IL1RAP* significantly associated with microglia activation. Therefore, further longitudinal GWAS on a series of QBs in the ADNI database are needed.

It is noteworthy that most of the previous GWAS of the AD were based on autosomes, and only a few considered X chromosome. Carrasquillo et al. (2009) analyzed late-onset AD in a case–control GWAS and found a significant SNP rs5984894, which is the first identified X-linked locus in the AD GWAS (Bertram and Tanzi, 2009). This SNP is located in the *PCDH11X* gene, which encodes a protocadherin, a cell–cell adhesion molecule expressed in the brain (Naj et al., 2010). Furthermore, Davis et al. (2021) identified 29 genes on the X chromosome were significantly associated with cognitive change. Among these X-linked genes, proteins encoded by *GRIA3*, *GPRASP2* and *GRIPAP1* (or *GRASP1*) are essential for synaptic transmission, plasticity mechanisms and cognitive substrates (Davis et al., 2021). Bajic et al. (2015) suggested that the AD has always been characterized by X chromosome instability, and its inactivation pattern is closely related to the pathogenesis of the AD (Bajic et al., 2020). Napolioni et al. performed an X chromosome-wide association study (XWAS) for late-onset AD in 12,987 Northwestern Europeans and discovered an X-linked locus rs112930037 on Xq25 *DCAF12L2*, which is widely expressed in the brain (Napolioni et al., 2017). Christopher et al. also conducted an XWAS and found that the SNP rs11094635 located upstream of the *MTM1* gene is associated with beta-amyloid accumulation in the ADNI database (Christopher et al., 2018), but ignored the studies of other QBs in the ADNI database. Note that so far, we are not aware of any XWAS for different types of QBs in the ADNI database, although there have been some previous GWAS based on autosomal loci (Li et al., 2017; Kong et al., 2018; Zhou et al., 2018; Wang et al., 2021; Homann et al., 2022; Oatman et al., 2023). Therefore, there is an urgent need to perform XWAS on the QBs of the ADNI. However, several analytical challenges arise from the complex biological mechanism of the X chromosome, including the differences of the copy number of the X chromosome between sexes and X chromosome inactivation (XCI) in females (Wu et al., 2014; Sauteraud et al., 2021). To balance the differences of transcriptional dosage between sexes, XCI transcriptionally silences one of the two X chromosomes in females during early embryogenesis (Smith et al., 2021). There are three patterns of XCI, random XCI (XCI-R), escape from XCI (XCI-E) and skewed XCI (Yu et al., 2022). The XCI-R is defined as the maternal or paternal alleles in females being expressed mono-allelically in different cell populations with probability approximately 50% (Wang et al., 2014; Jin et al., 2016). Additionally, approximately 15–30% of the X-linked genes are subject to undergo XCI-E, and express both alleles in female cells (Carrel et al., 2006; Posynick and Brown, 2019). Finally, the skewed XCI means that more than 75% of the cells in females have the same allele inactivated (Minks et al., 2008). For some extreme cases, it is possible that more than 90% of cells with the same allele inactive (Minks et al., 2008; Chabchoub et al., 2009). Accordingly, identifying the associations between the QBs of the AD and X-chromosomal SNPs requires special consideration. In recent years, to effectively incorporate the information of the XCI, some association analysis methods for the X-chromosomal SNPs have been developed (Chen et al., 2017, 2021; Özbek et al., 2018; Deng et al., 2019; Wang P. et al., 2019; Yang et al., 2022). Deng et al. (2019) proposed a two-stage method (i.e., wM3VNA3.3) to test the SNP effect on the phenotypic variances, while this method was not designed to test for the differences in the phenotypic means. Özbek et al. (2018) and Chen et al. (2021) respectively suggested an X chromosomal association test statistic to detect the SNP effect on the phenotypic means (respectively denoted by $T_{\mathrm{plink}}$ and $T_{\mathrm{chen}}$ in this article). Note that $T_{\mathrm{plink}}$ and $T_{\mathrm{chen}}$ only compare the difference in the phenotypic means across different genotypes under the assumption of variance homogeneity, which may lead to increasing false positive results in the presence of variance heterogeneity. Influencing factors of variance heterogeneity include genotype-by-environment interactions (Wang H. et al., 2019), XCI (Deng et al., 2019), etc. Therefore, Yang et al. (2022) proposed the weighted versions of $T_{\mathrm{plink}}$ and $T_{\mathrm{chen}}$ (i.e., $T_{\mathrm{plinkw}}$ and $T_{\mathrm{chenw}}$ ), which estimate the regression coefficients using the weighted least square method. Additionally, Yang et al. (2022) developed four novel X chromosome association analysis methods (i.e., QXcat , ${\mathrm{QZ}}_{\max}$ , QMVXcat , and ${\mathrm{QMVZ}}_{\max}$ ), all of which effectively take account of the information of the XCI. Among them, QXcat and ${\mathrm{QZ}}_{\max}$ were designed for testing the differences in the phenotypic means, while QMVXcat and ${\mathrm{QMVZ}}_{\max}$ can simultaneously test for both the phenotypic mean and variance differences, where combining the *p*-values is based on Fisher’s method (Fisher et al., 1967). However, all the above-mentioned methods are only applicable to cross-sectional studies. For longitudinal data, there is no specific method on the X chromosome available. The general practice is to assume that the XCI pattern is XCI-R or XCI-E, and then use the same analytical strategy as the autosomes to fit a linear mixed model (LMM).

Therefore, in this article, using the data from the ADNI database, we conducted XWAS between 16 QBs and X-chromosomal SNPs based on both the cross-sectional and longitudinal studies. The purpose of this article is to propose new insights into the molecular genetics behind the AD, and to provide a mechanistic clue to further AD-related studies.

## 2. Materials and methods

### 2.1. Samples

The data used in this article were downloaded and analyzed from the ADNI database^1^ (ADNI, 2022). The ADNI was launched in 2003 as a public-private partnership, led by Principal Investigator Michael W. Weiner, MD. The main goal of the ADNI has been always to test whether serial MRI, positron emission tomography (PET), other biological markers, and clinical and neuropsychological assessment can be combined to measure the progression of MCI and early AD (for the latest information see text footnote 1) (ADNI, 2022). As a multisite longitudinal study, the ADNI has recruited the subjects aged 55–90 years in North America and elsewhere since 2004. These subjects were from four ethnic groups, non-Hispanic White, non-Hispanic African American, Hispanic, and others (Wang et al., 2021). With the subjects’ informed consent, the ADNI conducted a series of initial tests on them and repeated the tests over subsequent years, including clinical evaluations, neuropsychological tests, genetic tests, and more. Currently, the ADNI has carried out four cohorts, ADNI cohorts 1, GO, 2 and 3 in sequence, sharing the data with researchers around the world and making a significant contribution to the AD research. Considering the small number of the subjects with genetic data for ADNI cohort 3 (only 327), we analyzed only the first three cohorts. In addition, we merged ADNI cohorts GO and 2 into one cohort, namely ADNI cohort GO/2, since their genotyping platforms were both the Illumina HumanOmniExpress BeadChip and their recorded genetic data were similar (Jack et al., 2015; Moore et al., 2019; St John-Williams et al., 2019). In a nutshell, 620,901 SNPs on both autosomes and X chromosome and 757 subjects were included in ADNI cohort 1, and 730,525 SNPs and 793 subjects were contained in ADNI cohort GO/2, which are two independent cohorts.

### 2.2. Genotyping and imputation

**Figure 1:**
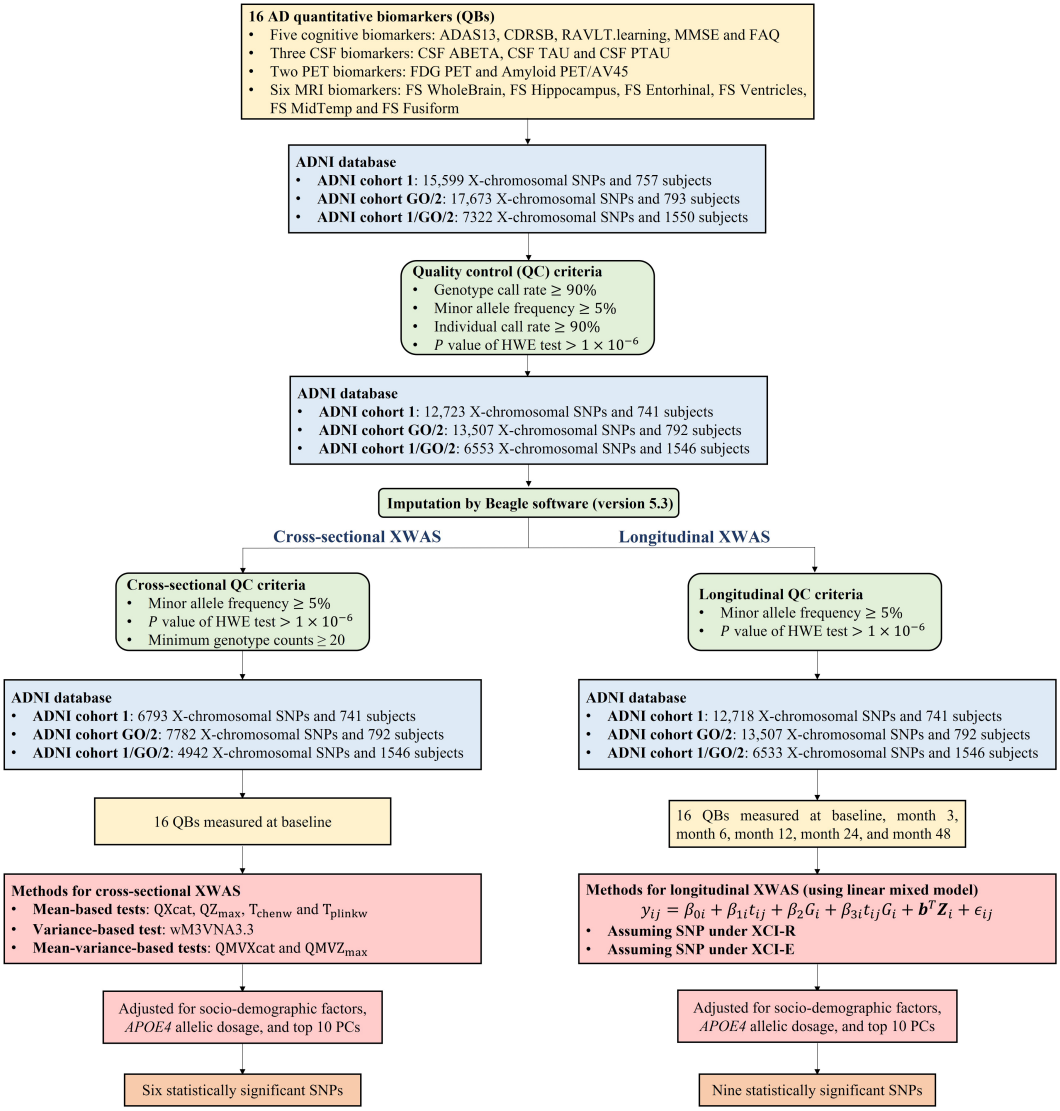
Workflow of the cross-sectional and longitudinal XWAS together with the quality control process and the number of identified SNPs. HWE, Hardy–Weinberg equilibrium; PC, principal component.

The genotyping platforms used in ADNI cohorts 1 and GO/2 were the Illumina Human610-Quad BeadChip and the Illumina HumanOmniExpress BeadChip, respectively. We selected 15,599 X-chromosomal SNPs in ADNI cohort 1 and 17,673 X-chromosomal SNPs in ADNI cohort GO/2, which are not in the pseudoautosomal region of the X chromosome. Note that in both the cohorts, there are 7,322 SNPs are overlapping (Supplementary Figure S1). Then, for longitudinal XWAS, we filtered these SNPs and the subjects by using the following quality control (QC) criteria (Özbek et al., 2018; Yang et al., 2022), in ADNI cohorts 1 and GO/2, respectively: (1) genotype call rate ≥90%, (2) minor allele frequency ≥5%, (3) individual call rate ≥90%, based on the SNPs on the X chromosome, and (4) the *p*-value of the Hardy–Weinberg equilibrium test $>1\times 10^{-6}$ . Here, the minor allele frequency at each SNP was estimated by $\left(2n_{AA,f}+n_{Aa,f}+n_{A,m}\right)/\left(2n_{f}+n_{m}\right)$ (Ma et al., 2015), where a and A are the major and minor alleles, respectively; $n_{f}$ and $n_{m}$ are the number of the females and that of the males, respectively; $n_{AA,f}$ and $n_{Aa,f}$ are the numbers of the females with genotypes AA and Aa , respectively; $n_{A,m}$ refers to the number of the males with allele A . QC criteria (1)–(3) were implemented in PLINK (version 1.9) (Purcell et al., 2007). Note that the Hardy–Weinberg equilibrium test in PLINK is carried out only based on female genotypes, while the exact test in the R package “HardyWeinberg” (Graffelman and Weir, 2016) can consider both the female and male genotypes. So, we used this R package for QC criterion (4). For each cohort, we imputed them using Beagle software (version 5.3) (Browning et al., 2018, 2021) without any reference panel, just based on the genotyped SNPs, and stratified by sexes according to its instruction, as females are diploids while males are hemizygotes in the non-pseudoautosomal region, when there are missing genotypes at the filtered SNPs. After the imputation, QC criteria (2) and (4) were conducted again in ADNI cohorts 1 and GO/2 separately. Figure 1 gives the detailed QC process for longitudinal XWAS. Finally, 12,718 X-chromosomal SNPs and 741 subjects were remained in ADNI cohort 1, while 13,507 SNPs X-chromosomal SNPs and 792 subjects were retained in ADNI cohort GO/2 (Figure 1). On the other hand, for cross-sectional XWAS, besides the aforementioned process (i.e., QC criteria (1)–(4), the imputation, and then QC criteria (2) and (4) again), we added an additional QC criterion to filter the SNPs, i.e., (5) the minimum genotype counts ≥20, to avoid the inflated type I errors (Soave et al., 2015). That is to say, only the SNPs which additionally meet criterion (5) were included in the cross-sectional XWAS. Lastly, the numbers of the X-chromosomal SNPs remained in ADNI cohorts 1 and GO/2 in the cross-sectional study were 6,793 and 7,782, and the corresponding sample sizes were 741 and 792, respectively (Figure 1). Note that different subjects may have missing values for different QBs. Thus, for different QBs, there may be different sample sizes and different numbers of the SNPs. The sample size and the number of the used SNPs for each QB and each ADNI cohort under the QC criteria (1)–(5) in the cross-sectional XWAS were listed in Supplementary Table S1. We analyzed all the respective filtered X-chromosomal SNPs in ADNI cohorts 1 and GO/2, including the overlapping SNPs in both of them. To further verify the results at the overlapping X-chromosomal SNPs in ADNI cohorts 1 and GO/2 and see if there are additional statistically significant SNPs to be found due to the increased sample size, we merged these two cohorts as a new cohort with 7,322 X-chromosomal SNPs and 1,550 subjects, namely ADNI cohort 1/GO/2 (Li et al., 2016; Lorenzi et al., 2018; Lee et al., 2022), because the QBs and the covariates in these two cohorts are the same. We conducted the process for ADNI cohort 1/GO/2 which is the same as ADNI cohorts 1 and GO/2 (the QC, the imputation and then the QC again). Eventually, 6,533 (4,942) SNPs on the X chromosome and 1,546 (1,546) subjects were kept in ADNI cohort 1/GO/2 for the longitudinal (cross-sectional) XWAS (Figure 1).

### 2.3. Clinical phenotypes

According to the analytical ideas of the Quantitative Template for the Progression of the AD Project^2^ (Portland Institute for Computational Science, 2022) recommended by several research groups (Jedynak et al., 2012; Donohue et al., 2014; Young et al., 2014; Schmidt-Richberg et al., 2015), we selected 16 QBs of the AD, which can reflect the main changes of the AD process across four modalities: cognitive assessment, CSF, PET, and MRI. Table 1 shows the full names and the corresponding abbreviations of these 16 QBs of the AD in detail.

**Table 1:** 16 QBs of AD in ADNI database.

Category	Full name	Abbreviation
Cognition	Alzheimer’s Disease Assessment Scale-Cognitive 13	ADAS13
Cognition	Clinical Dementia Rating-Sum of Boxes	CDRSB
Cognition	Rey Auditory Verbal Learning Test Learning	RAVLT.learning
Cognition	Mini-Mental State Exam	MMSE
Cognition	Functional Assessment Questionnaire	FAQ
CSF	Cerebrospinal Fluid Amyloid Beta 42	CSF ABETA
CSF	Cerebrospinal Fluid Tau	CSF TAU
CSF	Cerebrospinal Fluid Phosphorylated Tau	CSF PTAU
PET	Fluorodeoxyglucose Positron Emission Tomography	FDG PET
PET	Amyloid Positron Emission Tomography	Amyloid PET/AV45
MRI	FreeSurfer Whole Brain Volume	FS WholeBrain
MRI	FreeSurfer Hippocampus Volume	FS Hippocampus
MRI	FreeSurfer Entorhinal Cortex Volume	FS Entorhinal
MRI	FreeSurfer Ventricular Volume	FS Ventricles
MRI	FreeSurfer Middle Temporal Gyrus Volume	FS MidTemp
MRI	FreeSurfer Fusiform Volume	FS Fusiform

For cognitive assessment, five cognitive tests were chosen as cognitive biomarkers, including ADAS13, CDRSB, RAVLT.learning, MMSE, and FAQ. The ADAS13 scores the subjects with 13 subscales (Podhorna et al., 2016) to evaluate memory, reasoning, language, orientation, ideational praxis, constructional praxis, etc., and the test is scored in terms of errors, with higher scores indicating poorer performance. The CDRSB is a global rating of dementia which aggregates impairment in six categories of cognitive function, including memory, orientation, judgment and problem solving, community affairs, home and hobbies, and personal care. A higher score is indicative of more severe disease (Doody et al., 2010). The RAVLT is an episodic memory measure which requires subjects to perform a word learning trial and immediately recall it. This process is repeated 5 times (Trials 1–5), resulting in the scores for five trials. And the RAVLT.learning is the score of Trial 5 minus that of Trial 1. The MMSE is a 30-item cognitive state assessment (Chapman et al., 2016) involving orientation, memory, attention, concentration, naming, repetition, comprehension and other abilities. It is scored as the number of the items completed correctly, and lower score represents poor performance and more severe cognitive impairment. The FAQ rates the subjects’ disability to perform 10 activities of daily living and the scores of 10 activities are then added together to provide a total disability score.

The three core CSF biomarkers related to the AD involved in our analysis are CSF ABETA, CSF TAU, and CSF PTAU (Babić et al., 2014). The CSF ABETA and the CSF TAU, respectively, reflect the deposition of amyloid in the cerebral cortex and the density of neurodegeneration, and the CSF PTAU is related to the pathological changes of neurofibrillary tangles. The decreased level of the ABETA and the elevated levels of the TAU and the PTAU are the important CSF features of the AD (Zou et al., 2020).

Neuroimaging data used in this article included two PET and six MRI biomarkers. Specifically, the FDG PET and Amyloid PET/AV45 measurements are captured using PET image acquisition techniques. The former can reflect the extent of cellular metabolism in brain regions, and if this region is affected by the AD, it indicates a decrease in metabolism (Hojjati and Babajani-Feremi, 2022), while the latter assesses the amyloid load in the brain associated with the AD (Saint-Aubert et al., 2013). The average FDG PET of angular gyrus, temporal gyrus, and posterior cingulate cortex, and the mean florbetapir (Amyloid PET/AV45) of the whole cerebellum can be obtained from the ADNI. More details please refer to the description about the PET analysis on the ADNI website (see text footnote 1) (ADNI, 2022). Moreover, the MRI biomarkers (FS WholeBrain, FS Hippocampus, FS Entorhinal, FS Ventricles, FS MidTemp, and FS Fusiform) are the volumes of whole brain, hippocampus, entorhinal cortex, ventricles, middle temporal gyrus and fusiform gyrus obtained from the FreeSurfer software. More details on the MRI data can be found elsewhere (Jack et al., 2008; Wyman et al., 2013; Weiner et al., 2015).

Before the XWAS, we conducted the normality tests of 16 QBs at the baseline and the correlation tests among them. Initially, we drew the Q-Q plots (Supplementary Figures S2–S4) and carried out the Shapiro–Wilk tests for the normality at the significance level of $0.05/16=3.13\times 10^{-3}$ after Bonferroni correction (Supplementary Table S2). As shown in Supplementary Figures S2–S4 and Supplementary Table S2, we found that FS WholeBrain, FS Hippocampus, FS Entorhinal, FS MidTemp and FS Fusiform asymptotically follow the normal distributions, while other 11 QBs appear not to satisfy the normality assumptions. Thus, we used the rank-based inverse normal transformation proposed by McCaw et al. (2020) to transform these 11 QBs in the subsequent analyses (i.e., ADAS13, CDRSB, RAVLT.learning, MMSE, FAQ, CSF ABETA, CSF TAU, CSF PTAU, FDG PET, Amyloid PET/AV45, and FS Ventricles). Furthermore, we computed the Pearson’s correlation coefficients among FS WholeBrain, FS Hippocampus, FS Entorhinal, FS MidTemp and FS Fusiform, while all other possible pairs of the QBs were analyzed with the Spearman’s rank correlation coefficients (see Supplementary Figures S5–S7 for the details). It is shown in Supplementary Figures S5–S7 that the correlations in ADNI cohort 1 are generally consistent with those in ADNI cohort GO/2, and most of the QBs are significantly correlated (*p*-values less than $4.76\times 10^{-4}$ in ADNI cohort 1 and $4.17\times 10^{-4}$ in ADNI cohort GO/2, respectively based on Bonferroni correction for 105 and 120 tests).

### 2.4. Covariates

To deal with the population stratification caused by different races and other factors, we used PLINK (version 1.9) (Purcell et al., 2007) to perform the principal component analysis (Price et al., 2010) of all the autosomal and X-chromosomal SNPs, and chose the top 10 principal components (PCs) generally recommended (Zhao et al., 2018). Besides, socio-demographic factors are required to be adjusted, include sex, age and educational level. Education level is defined by years of education. Therefore, all the analyses in this article were implemented by adjusting socio-demographic factors, *APOE4* allelic dosage (i.e., the number of ε4 alleles in a subject’s *APOE* genotype), and the top 10 PCs. For ADNI cohort 1/GO/2, besides the above covariates (i.e., sex, age, education level, *APOE4* allelic dosage and the top 10 PCs), another covariate (i.e., batch) is included as a dummy variable to adjust the effect of different genotyping batches.

### 2.5. Cross-sectional XWAS

In the cross-sectional XWAS, we used the methods testing for the difference in the means of the QBs (i.e., QXcat , ${\mathrm{QZ}}_{\max}$ , $T_{\mathrm{chenw}}$ , and $T_{\mathrm{plinkw}}$ ) (Yang et al., 2022), the method testing for the difference in the variances of the QBs (i.e., wM3VNA3.3) (Deng et al., 2019), and the methods simultaneously testing for the mean and variance differences of the QBs (i.e., ${\mathrm{QMVX}}_{\mathrm{cat}}$ and ${\mathrm{QMVZ}}_{\max}$ ) (Yang et al., 2022), to detect the association between each of the 16 QBs at the baseline and the X-chromosomal SNPs. Note that the test statistics QXcat , ${\mathrm{QZ}}_{\max}$ , ${\mathrm{QMVX}}_{\mathrm{cat}}$ , and ${\mathrm{QMVZ}}_{\max}$ were constructed by stratifying the collected sample into females and males. So, the two coding schemes G={0,1} and {0,2} for the male genotypes have no influence on the results. More details about these methods are given in Supplementary methods. The Bonferroni correction for multiple comparison was utilized and the corrected significance level of the association tests was set to be $0.05/19692=2.54\times 10^{-6}$ in ADNI cohorts 1, GO/2 and 1/GO/2, where 19,692 is the number of the X-chromosomal SNPs after the QC used in this article, consisting of 12,718 SNPs in ADNI cohort 1 and 13,507 SNPs in ADNI cohort GO/2, with the number of the overlapping SNPs being 6,533 in ADNI cohort 1/GO/2.

### 2.6. Longitudinal XWAS

To examine the longitudinal effects of the SNPs on the X chromosome, we used the LMM to model each of the 16 longitudinal QBs separately. Despite the extensive longitudinal data with 35 visits provided in the ADNI, not all the 16 QBs were simultaneously recorded for every subject at each visit. As such, the subjects who had at least one measurement were included in our analysis, and there were fewer data available as the follow-up progressed. Therefore, to ensure that the LMM has the sufficient statistical power, we only studied six visits with at least 200 subjects being followed up, namely baseline (bl), month 3 (m03), month 6 (m06), month 12 (m12), month 24 (m24), and month 48 (m48) visits.

Given the specific characteristics of the X chromosome, although we can code the female genotypes as the number of the minor alleles under an additive model (i.e., G={0,1,2} ), as we do for autosomal SNPs, there are two distinct coding schemes for the male genotypes. Under the XCI-R, where one of the X chromosomes in females is inactive, one male minor allele is equivalent to the corresponding female homozygote, so we coded the male genotype as G={0,2} . On the other hand, under the XCI-E, where both the X chromosomes in females are expressed, we coded the male genotype as G={0,1} (Ma et al., 2015). For each X-chromosomal SNP, the LMM can be expressed as follows


(1) $$y_{ij}=\beta_{0i}+\beta_{1i}t_{ij}+\beta_{2}G_{i}+\beta_{3i}t_{ij}G_{i}+b^{T}Z_{i}+\in_{ij},$$

where $y_{ij}$ is the original or transformed QB of subject *i* at visit *j*, i=1,2,…,N;j=1,2,…,6, and *N* is the sample size; $t_{ij}$ is the visit time, which was coded as 1, 2, …, 6 for the six visits, respectively; $G_{i}$ denotes the genotype code at the SNP of interest for subject i ; $t_{ij}G_{i}$ is the time × SNP interaction term; $Z_{i}$ is a vector of the covariates (i.e., sex, age, education level, *APOE4* allelic dosage, the top 10 PCs and the batch) for subject i . $\beta_{0i}$ is the random intercept for subject i ; $\beta_{1i}$ is the random effect for time $t_{ij}$ ; $\beta_{2}$ is the regression coefficient of the genotype code $G_{i}$ ; $\beta_{3i}$ is the random effect for the interaction term $t_{ij}G_{i}$ ; ***b*** is a vector of the regression coefficients of the covariates $Z_{i}$ ; $\epsilon_{ij}$ is a random error which follows $N\left(0,\sigma_{e}^{2}\right)$ , where $\sigma_{e}^{2}$ is the variance of $\epsilon_{ij}$ and we assumed that the variances of $\epsilon_{ij}$ across different genotypes at the SNP are the same.

In the longitudinal XWAS, we investigated whether or not the SNP affects the rate of the change of each of the original or transformed QBs, so the effect estimate of interest is that of the time×SNP interaction (i.e., $\beta_{3i}$ ), which represents the longitudinal effect of the SNP (Sikorska et al., 2015; Carrion-Castillo et al., 2017; Allen et al., 2023). Just like the cross-sectional XWAS, we used the Bonferroni correction to take account of multiple testing and fixed the corrected significance level of the association tests in ADNI cohorts 1, GO/2 and 1/GO/2 to be $2.54\times 10^{-6}$ here.

### 2.7. Implementation in R

All statistical analyses were conducted in the R software (version 4.1.2) (Team, 2013). The cross-sectional XWAS was fitted with the R package “QMVtest” (Yang et al., 2022). The LMM was fitted with the R package “lme4” (Bates et al., 2015) and the *p*-values were derived using the Satterthwaite approximation of the “lmerTest” package (Kuznetsova et al., 2017).

## 3. Results

### 3.1. Characteristics of subjects

From Figure 1, there are 757 and 793 subjects in ADNI cohorts 1 and GO/2, respectively, and the sample size of the merged ADNI cohort 1/GO/2 is 1,550. After the QC, ADNI cohorts 1, GO/2, and 1/GO/2 contain 741, 792, and 1,546 subjects, respectively. Table 2 presents the baseline characteristics of the subjects in ADNI cohorts 1, GO/2, and 1/GO/2. For all the 1,546 subjects in ADNI cohort 1/GO/2, the median age is 74.00 years and the interquartile range (IQR) is 69.33 ~ 78.97 years, and 676 (43.73%) are females and 870 (56.27%) are males. The majority of the subjects (1,384) are non-Hispanic White (89.52%), followed by 69 non-Hispanic African American (4.46%), 51 Hispanic (3.30%), and 42 others (2.72%). In addition, ADNI cohort GO/2 has more women, a younger median age, and lower *APOE4* allelic dosage than ADNI cohort 1. As for 15 QBs (the values of Amyloid PET/AV45 at baseline are missing in ADNI cohort 1), compared to ADNI cohort 1, ADNI cohort GO/2 has lower ADAS13, CDRSB, FAQ, CSF TAU, CSF PTAU, and FS Ventricles, while the remaining QBs (RAVLT.learning, MMSE, CSF ABETA, FDG PET, FS WholeBrain, FS Hippocampus, FS Entorhinal, FS MidTemp, and FS Fusiform) are higher.

**Table 2:** Baseline characteristics of the subjects in ADNI cohorts 1, GO/2, and 1/GO/2.

Variable	ADNI cohort 1 (*N* = 741)	ADNI cohort GO/2 (*N* = 792)	ADNI cohort 1/GO/2 (*N* = 1,546)
Sex			
Female, No. (%)	308 (41.57)	368 (46.46)	676 (43.73)
Male, No. (%)	433 (58.43)	424 (53.54)	870 (56.27)
Age (in years), median (IQR)	75.50 (71.20 ~ 80.00)	72.60 (67.57 ~ 77.70)	74.00 (69.33 ~ 78.97)
Education level (in years), median (IQR)	16 (13 ~ 18)	16 (14 ~ 18)	16 (14 ~ 18)
*APOE4* allelic dosage			
0, No. (%)	373 (50.34)	439 (55.43)	817 (52.85)
1, No. (%)	290 (39.14)	283 (35.73)	579 (37.45)
2, No. (%)	78 (10.53)	70 (8.84)	150 (9.70)
Race			
White, No. (%)	671 (90.55)	701 (88.51)	1,384 (89.52)
African American, No. (%)	37 (4.99)	32 (4.04)	69 (4.46)
Hispanic, No. (%)	17 (2.29)	34 (4.29)	51 (3.30)
Others, No. (%)	16 (2.16)	25 (3.16)	42 (2.72)
ADAS13 (in scores), median (IQR)	18.00 (11.00 ~ 24.33)	13.00 (8.00 ~ 20.00)	15.00 (9.67 ~ 23.00)
CDRSB (in scores), median (IQR)	1.5 (0 ~ 3.0)	1.0 (0 ~ 2.0)	1.0 (0 ~ 2.5)
RAVLT.learning (in scores), median (IQR)	3 (2 ~ 6)	4 (3 ~ 7)	4 (2 ~ 6)
MMSE (in scores), median (IQR)	27 (25 ~ 29)	29 (26 ~ 30)	28 (26 ~ 29)
FAQ (in scores), median (IQR)	2.00 (0 ~ 8.00)	1.00 (0 ~ 5.00)	1.00 (0 ~ 6.00)
CSF ABETA (in pg./mL), median (IQR)	694.15 (535.88 ~ 1229.25)	928.75 (664.42 ~ 1532.50)	858.70 (596.60 ~ 1406.00)
CSF TAU (in pg./mL), median (IQR)	281.45 (209.85 ~ 368.45)	246.75 (186.88 ~ 329.82)	257.80 (193.50 ~ 351.80)
CSF PTAU (in pg./mL), median (IQR)	27.18 (18.65 ~ 37.13)	22.64 (16.74 ~ 32.22)	24.08 (17.31 ~ 34.05)
FDG PET (in SUVR), median (IQR)	1.24 (1.18 ~ 1.29)	1.27 (1.22 ~ 1.32)	1.26 (1.21 ~ 1.31)
Amyloid PET/AV45^a^ (in SUVR), median (IQR)	–	1.13 (1.02 ~ 1.40)	1.13 (1.02 ~ 1.40)
FS WholeBrain (in mm^3^), mean ± SD^b^	989673.62 ± 106621.05	1048929.29 ± 105742.11	1020009.00 ± 110047.70
FS Hippocampus (in mm^3^), mean ± SD^b^	6472.01 ± 1169.68	7036.37 ± 1127.23	6778.00 ± 1181.27
FS Entorhinal (in mm^3^), mean ± SD^b^	3332.98 ± 809.55	3632.71 ± 729.62	3491.00 ± 782.89
FS Ventricles (in mm^3^), median (IQR)	38509.00 (26164.75 ~ 55523.50)	32916.00 (21764.00 ~ 48505.00)	35549.00 (24229.00 ~ 51527.00)
FS MidTemp (in mm^3^), mean ± SD^b^	18624.48 ± 3109.97	20157.52 ± 2834.14	19438.00 ± 3056.10
FS Fusiform (in mm^3^), mean ± SD^b^	16191.48 ± 2480.94	18226.60 ± 2630.74	17273.00 ± 2755.35

IQR, interquartile range; pg/mL, picograms per milliliter; SD, standard deviation; SUVR, standardized uptake volume ratio. ^a^The values of Amyloid PET/AV45 at baseline are missing in ADNI cohort 1. ^b^The descriptive characteristics of FS WholeBrain, FS Hippocampus, FS Entorhinal, FS MidTemp and FS Fusiform are presented as mean ± SD, since they asymptotically follow the normal distributions.

For the longitudinal data, the follow-up information for the two cohorts decreases with the follow-up proceeds, and the missing rates of the 16 QBs are also different (Supplementary Tables S3–S5). For example, none of the 16 QBs were measured at the month 3 visit in ADNI cohort 1; Amyloid PET/AV45 was not measured at the first 5 visits and was only measured at the month 6 visit in ADNI cohort 1; all the QBs of the cognition, the CSF and the PET were not recorded at the month 3 visit in ADNI cohort GO/2. Supplementary Figures S8–S10 give the spaghetti plots of the longitudinal course of the 16 QBs in ADNI cohort 1, GO/2, and 1/GO/2, respectively. As shown in Supplementary Figures S8–S10, the trajectories of the subjects corresponding to different QBs are highly variable. Therefore, it may be more appropriate to use the LMM to carry out the longitudinal XWAS, which can capture more information.

### 3.2. Cross-sectional XWAS

In the cross-sectional XWAS, we identified six SNPs (rs5927116, rs4596772, rs5929538, rs2213488, rs5920524, and rs5945306) which are statistically significantly associated with one of the QBs of the AD. Table 3 shows the *p*-values of all the methods ( ${\mathrm{QMVX}}_{\mathrm{cat}}$ , ${\mathrm{QMVZ}}_{\max}$ , QXcat , ${\mathrm{QZ}}_{\max}$ , $T_{\mathrm{chenw}}$ , $T_{\mathrm{plinkw}}$ , and wM3VNA3.3) at these six SNPs. Table 4 presents the positions, the major alleles, the minor alleles, the minor allele frequencies, the *p*-values of the Hardy–Weinberg equilibrium tests and the genes consisting of these six identified SNPs. SNP rs5927116 is in the *DMD* gene, which has the effects on the mean values of the FS Entorhinal ( $p_{\mathrm{QXcat}}=1.74\times 10^{-6}$ ). SNP rs4596772, located near to the *TBX22* gene, only influences the mean values of the FS MidTemp ( $p_{\mathrm{QXcat}}=9.94\times 10^{-7}$ and $p_{{\mathrm{QZ}}_{\max}}=7.55\times 10^{-7}$ ). SNP rs5929538 is included in the *LOC101928437* gene and has the effects on the mean values of the transformed FDG PET ( $p_{T_{\mathrm{chenw}}}=2.28\times 10^{-6}$ ). It is worth noting that SNP rs2213488, located in the *TENM1* gene, is an overlapping variant in both ADNI cohorts 1 and GO/2. However, it demonstrates the statistical significance only in the analysis of ADNI cohort GO/2 with 792 subjects ( $p_{{\mathrm{QMVX}}_{\mathrm{cat}}}=1.30\times 10^{-6}$ and $p_{{\mathrm{QMVZ}}_{\max}}=7.23\times 10^{-7}$ ), while it is not statistically significant in the analysis of ADNI cohort 1/GO/2 with 1,546 subjects ( $p_{{\mathrm{QMVX}}_{\mathrm{cat}}}=6.16\times 10^{-5}$ , $p_{{\mathrm{QMVZ}}_{\max}}=1.13\times 10^{-4}$ , $p_{\mathrm{QXcat}}=5.06\times 10^{-4}$ , $p_{{\mathrm{QZ}}_{\max}}=9.77\times 10^{-4}$ , $p_{T_{\mathrm{chenw}}}=7.86\times 10^{-4}$ , $p_{T_{\mathrm{plinkw}}}=3.56\times 10^{-4}$ , and $p_{\mathrm{wM}3\mathrm{VNA}3.3}=9.17\times 10^{-3}$ ). Moreover, from the *p*-values of all the methods for SNP rs2213488 in the analysis of ADNI cohort GO/2, only the *p*-values of ${\mathrm{QMVX}}_{\mathrm{cat}}$ and ${\mathrm{QMVZ}}_{\max}$ for simultaneously testing for the means and the variances of the FS Hippocampus are lower than the significance level $2.54\times 10^{-6}$ . This suggests that either the means or the variances of the FS Hippocampus across different genotypes are different, which needs to be further investigated. SNP rs5920524, located near to the *SPANXN1* gene, has the effects on the mean values of the transformed FDG PET ( $p_{\mathrm{QXcat}}=5.57\times 10^{-7}$ and $p_{T_{\mathrm{chenw}}}=5.97\times 10^{-7}$ ), and the resulting *p*-value of ${\mathrm{QMVX}}_{\mathrm{cat}}$ is $1.72\times 10^{-6}$ . Finally, SNP rs5945306, located in the *ZFP92* gene, only influences the mean values of the transformed FAQ ( $p_{\mathrm{QXcat}}=7.67\times 10^{-7}$ and $p_{T_{\mathrm{chenw}}}=9.22\times 10^{-8}$ ), and the resulting *p*-value of ${\mathrm{QMVX}}_{\mathrm{cat}}$ is $1.82\times 10^{-6}$ . On the other hand, to infer the direction of the SNP effect on the means and the variances of the QB under study, we can, respectively, observe the signs of the regression coefficients in the mean-based tests ( QXcat and ${\mathrm{QZ}}_{\max}$ ) and those in the variance-based test (wM3VNA3.3), and the corresponding detailed descriptions can be found in Supplementary results as well as Supplementary Tables S6, S7. For comparison, the results of the longitudinal XWAS for these six SNPs found by the cross-sectional XWAS are also shown in Supplementary Tables S8, S9, although they are not statistically significant.

**Table 3:** *p*-values of all the methods at six SNPs in cross-sectional XWAS.^a^

SNP	Trait	ADNI cohort	QMVX_cat_	QMVZ_max_	QX_cat_	QZ_max_	T_chenw_	T_plinkw_	wM3VNA3.3
rs5927116	FS entorhinal	GO/2	1.35 × 10^−5^	2.04 × 10^−5^	**1.74 × 10**^**−6**^	2.71 × 10^−6^	2.28 × 10^−5^	1.18 × 10^−4^	5.20 × 10^−1^
rs4596772	FS MidTemp	1	1.39 × 10^−5^	1.07 × 10^−5^	**9.94 × 10**^**−7**^	**7.75 × 10**^**−7**^	2.28 × 10^−5^	4.38 × 10^−5^	9.37 × 10^−1^
rs5929538	FDG PET^c^	1	2.39 × 10^−4^	1.15 × 10^−1^	6.05 × 10^−5^	7.34 × 10^−2^	**2.28 × 10**^**−6**^	1.06 × 10^−3^	3.34 × 10^−1^
rs2213488^b^	FS Hippocampus	GO/2	**1.30 × 10**^**−6**^	**7.23 × 10**^**−7**^	3.70 × 10^−4^	1.99 × 10^−4^	8.20 × 10^−4^	2.39 × 10^−4^	2.02 × 10^−4^
rs5920524	FDG PET^c^	GO/2	**1.72 × 10**^**−6**^	6.06 × 10^−6^	**5.57 × 10**^**−7**^	2.12 × 10^−6^	**5.97 × 10**^**−7**^	3.82 × 10^−6^	1.81 × 10^−1^
rs5945306	FAQ^c^	1/GO/2	**1.82 × 10**^**−6**^	9.42 × 10^−5^	**7.67 × 10**^**−7**^	5.29 × 10^−5^	**9.22 × 10**^**−8**^	4.17 × 10^−6^	1.39 × 10^−1^

^a^The *p*-values less than the significance level of 2.54 ×10^–6^ are highlighted in bold. ^b^SNP rs2213488 is an overlapping variant in both ADNI cohorts 1 and GO/2. However, it demonstrates the statistical significance only in the analysis of ADNI cohort GO/2 with 792 subjects, while it is not statistically significant in the analysis of ADNI cohort 1/GO/2 with 1,546 subjects ( $p_{{\mathrm{QMVX}}_{\mathrm{cat}}}=6.16\times 10^{-5},$ $p_{{\mathrm{QMVZ}}_{\max}}=1.13\times 10^{-4},$ $p_{\mathrm{QXcat}}=5.06\times 10^{-4},$ $p_{{\mathrm{QZ}}_{\max}}=9.77\times 10^{-4},$ $p_{T_{\mathrm{chenw}}}=7.86\times 10^{-4},$ $p_{T_{\mathrm{plinkw}}}=3.56\times 10^{-4}$ and $p_{\mathrm{wM}3\mathrm{VNA}3.3}=9.17\times 10^{-3}$ ). ^c^FDG PET and FAQ are transformed using the rank-based inverse normal transformation.

**Table 4:** Information on 15 SNPs identified in cross-sectional XWAS and longitudinal XWAS.

XWAS	SNP	Position	Allele		MAF	*p-*value of HWE test	Gene
			Major	Minor			
Cross-sectional	rs5927116	32,974,359	*C*	*T*	0.450	0.593	*DMD*
	rs4596772	79,847,546	*G*	*A*	0.377	0.211	Near to *TBX22*
	rs5929538	113,049,595	*G*	*A*	0.485	0.485	*LOC101928437*
	rs2213488^a^	125,142,940	*C*	*T*	0.255	0.208	*TENM1*
	rs5920524	145,431,897	*C*	*T*	0.500	0.996	Near to *SPANXN1*
	rs5945306	153,412,601	*T*	*C*	0.198	0.392	*ZFP92*
Longitudinal	rs12157031	20,663,243	*C*	*T*	0.072	0.606	*LOC124905257*
	rs428303	97,936,863	*G*	*A*	0.365	0.014	Near to *NCKAP1P1*
	rs4829868	137,246,548	*G*	*T*	0.085	0.116	Near to *RAC1P4*
	rs5931111	137,258,892	*C*	*T*	0.091	3.112 × 10^−4^	Near to *RAC1P4*
	rs5953487	140,548,099	*T*	*C*	0.333	0.014	Near to *LOC105373344*
	rs10284107	143,202,859	*A*	*G*	0.226	0.747	Near to *RN7SKP149*
	rs5955016	143,402,732	*T*	*C*	0.430	0.789	Near to *MTND1P33*
	rs6540385	148,580,366	*T*	*C*	0.428	0.090	*AFF2*
	rs763320	148,593,511	*T*	*G*	0.433	0.134	*AFF2*

HWE, Hardy–Weinberg equilibrium; MAF, minor allele frequency. ^a^For SNP rs2213488, the estimation of the MAF and the calculation of the *p*-value of the HWE test were based on ADNI cohort GO/2 with 792 subjects.

### 3.3. Longitudinal XWAS

We tested the longitudinal effects of 19,692 X-chromosomal SNPs on the 16 QBs of the AD by model (1) under the assumption that the XCI pattern of the SNP under study is the XCI-R and the XCI-E, respectively. We identified nine SNPs which show the statistically significant effects of the time×SNP interaction at the significance level $2.54\times 10^{-6}$ (rs12157031, rs428303, rs4829868, rs5931111, rs5953487, rs10284107, rs5955016, rs6540385, and rs763320), where SNP rs12157031 is statistically significantly associated with the original FS MidTemp in ADNI cohort 1/GO/2, SNP rs5931111 has the statistically significant effect on the transformed FS Ventricles in ADNI cohort 1, and other seven SNPs have statistically significant effects on the transformed FS Ventricles in ADNI cohort 1/GO/2. Table 5 and Supplementary Table S10 list the estimates of the time×SNP interaction effects and the SNP main effects, respectively. In Table 4, we also gave the positions, the major alleles, the minor alleles, the minor allele frequencies, the *p*-values of the Hardy–Weinberg equilibrium test, and the genes consisting of these nine identified SNPs. Specifically, SNP rs12157031 within the *LOC124905257* gene has a time×SNP interaction effect of 59.803 on the original FS MidTemp under the XCI-R and the corresponding 95% confidence interval (CI) is 36.738 ~ 82.867 ( $p=7.73\times 10^{-7}$ ). The minor allele T at rs12157031 increases the FS MidTemp over time. As for the eight SNPs associated with the transformed FS Ventricles, they all show significant time × SNP interaction effects under the XCI-E. The minor allele A at SNP rs428303 has the significant time × SNP interaction effect of −0.010 (95% CI: −0.014 ~ −0.006), with the corresponding *p*-value being $1.29\times 10^{-6}$ , decreasing the transformed FS Ventricles over time. The minor allele T at SNPs rs4829868 and rs5931111 shows significant time × SNP interaction effects of −0.017 (95% CI: −0.023 ~ −0.011; $p=3.19\times 10^{-8}$ ) and − 0.022 (95% CI: −0.030 ~ −0.015; $p=1.92\times 10^{-8}$ ), respectively, decreasing the transformed FS Ventricles over time. For SNP rs5953487, the regression coefficient of the interaction term is −0.010 (95% CI: −0.014 ~ −0.006; $p=1.50\times 10^{-6}$ ), which means that the minor allele C at rs5953487 reduces the transformed FS Ventricles over time. The minor alleles at SNPs rs10284107 and rs5955016 are, respectively, G and C, with the time × SNP interaction effects on the transformed FS Ventricles being, respectively, −0.012 (95% CI: −0.017 ~ −0.007; $p=1.19\times 10^{-6}$ ) and − 0.010 (95% CI: −0.014 ~ −0.006; $p=1.29\times 10^{-6}$ ), decreasing the transformed FS Ventricles over time. In addition, SNPs rs6540385 and rs763320 located in the *AFF2* gene, with the minor alleles C and G, respectively, both have the significant time × SNP interaction effect of −0.010 (95% CI: −0.014 ~ −0.006) with the respective *p*-values being $1.19\times 10^{-6}$ and $5.37\times 10^{-7}$ . This appears to demonstrate that these two SNPs would decrease the transformed FS Ventricles over time. On the other hand, we could not find any SNP main effect in the LMM (Supplementary Table S10). For comparison, the results of the cross-sectional XWAS for these nine SNPs detected by the longitudinal XWAS are also displayed in Supplementary Table S11, although they are not statistically significant.

**Table 5:** Estimates of time × SNP interaction effects at nine SNPs identified in longitudinal XWAS.

SNP	Trait	ADNI cohort	XCI-R			XCI-E		
			Time × SNP	95% CI	*p*-value^b^	Time × SNP	95% CI	*p*-value^b^
rs12157031	FS MidTemp	1/GO/2	59.803	36.738 ~ 82.867	**7.73 × 10**^**−7**^	89.681	52.564 ~ 126.797	3.82 × 10^−6^
rs428303	FS Ventricles^a^	1/GO/2	−0.006	−0.009 ~ −0.002	6.61 × 10^−4^	−0.010	−0.014 ~ −0.006	**1.29 × 10**^**−6**^
rs4829868	FS Ventricles^a^	1/GO/2	−0.010	−0.015 ~ −0.006	1.42 × 10^−5^	−0.017	−0.023 ~ −0.011	**3.19 × 10**^**−8**^
rs5931111	FS Ventricles^a^	1	−0.014	−0.020 ~ −0.008	9.80 × 10^−6^	−0.022	−0.030 ~ −0.015	**1.92 × 10**^**−8**^
rs5953487	FS Ventricles^a^	1/GO/2	−0.005	−0.008 ~ −0.001	4.14 × 10^−3^	−0.010	−0.014 ~ −0.006	**1.50 × 10**^**−6**^
rs10284107	FS Ventricles^a^	1/GO/2	−0.006	−0.010 ~ −0.003	3.20 × 10^−4^	−0.012	−0.017 ~ −0.007	**1.19 × 10**^**−6**^
rs5955016	FS Ventricles^a^	1/GO/2	−0.005	−0.008 ~ −0.002	3.82 × 10^−3^	−0.010	−0.014 ~ −0.006	**1.29 × 10**^**−6**^
rs6540385	FS Ventricles^a^	1/GO/2	−0.006	−0.009 ~ −0.003	1.26 × 10^−4^	−0.010	−0.014 ~ −0.006	**1.19 × 10**^**−6**^
rs763320	FS Ventricles^a^	1/GO/2	−0.006	−0.009 ~ −0.003	9.29 × 10^−5^	−0.010	−0.014 ~ −0.006	**5.37 × 10**^**−7**^

CI, confidence interval. ^a^FS Ventricles is transformed using the rank-based inverse normal transformation. ^b^The *p*-values less than the significance level of 2.54 ×10^–6^ are highlighted in bold.

## 4. Discussion

AD is a highly heritable disease which brings severe social, psychological, and economic burdens to patients (Andrews et al., 2020). However, most studies have only focused on autosomes, and relatively few studies have tested the susceptibility loci of the AD on the X chromosome. Leitão et al. (2022) undertook a systematic analysis of human genes on the X chromosome, and observed a higher proportion of disorder-associated genes and an enrichment of the genes on the X chromosome involved in cognition, language, and seizures, compared to autosomes. Therefore, in this article, we identified possible susceptibility loci for the 16 QBs of the AD in the ADNI database from both the cross-sectional and longitudinal perspectives. To the best of our knowledge, this is the first XWAS of the 16 key QBs of the AD for the ADNI database, including the cognitive, CSF, and neuroimaging QBs. Specifically, we noticed that the 11 QBs (i.e., ADAS13, CDRSB, RAVLT.learning, MMSE, FAQ, CSF ABETA, CSF TAU, CSF PTAU, FDG PET, Amyloid PET/AV45, and FS Ventricles) are not normally distributed. As such, we used the rank-based inverse normal transformation on these QBs before conducting the XWAS to reduce the false positive results. For the cross-sectional studies, we applied the methods testing for means (i.e., QXcat , ${\mathrm{QZ}}_{\max}$ , $T_{\mathrm{chenw}}$ , and $T_{\mathrm{plinkw}}$ ), the method testing for variances (i.e., wM3VNA3.3), and the methods simultaneously testing for means and variances ( QMVXcat and ${\mathrm{QMVZ}}_{\max}$ ) for analysis. Then, the LMM was utilized to carry out the longitudinal studies, assuming that the XCI pattern at the SNP under study is either the XCI-R or the XCI-E. Finally, 15 X-chromosomal SNPs were identified to be statistically significantly associated with one of the QBs of the AD. To clearly understand the whole article, Figure 1 gives the workflow of the cross-sectional and longitudinal XWAS, together with the corresponding QC process and the number of eventually identified SNPs.

Among the 15 identified SNPs, six SNPs were discovered by the cross-sectional XWAS, and nine SNPs were found by the longitudinal XWAS. These 15 SNPs are statistically significantly associated with one cognitive (i.e., FAQ), one PET (i.e., FDG PET) and four MRI (i.e., FS Hippocampus, FS Entorhinal, FS Ventricles, and FS MidTemp) biomarkers, where PET and MRI are neuroimaging biomarkers. We performed the functional annotation for these 15 SNPs through the following four databases: Genotype-Tissue Expression^3^ (GTEx, 2023), Genome Browser^4^ (Genome Browser, 2023), National Center for Biotechnology Information^5^ (NCBI, 2023) and GeneCards^6^ (GeneCards, 2023). Note that two of these SNPs, rs4829868 and rs5931111, which exhibit statistically significant time×SNP interaction effects on the FreeSurfer ventricular volume (i.e., FS Ventricles) in the longitudinal XWAS, with the respective *p*-values being $3.19\times 10^{-8}$ and $\;1.92\times 10^{-8}$ (both less than the genome-wide significance level of $5\times 10^{-8}$ ), are located near to the *RAC1P4* gene. The *RAC1P4* gene has been demonstrated to be associated with brain volume measurement (Smith et al., 2021). For the six SNPs (rs5927116, rs4596772, rs5929538, rs2213488, rs5920524, and rs5945306) identified in the cross-sectional XWAS, SNP rs5927116, belonging to the *DMD* gene, is statistically significantly associated with the volume of the entorhinal cortex (i.e., FS Entorhinal). The small entorhinal cortex volume has been proved to be an early predictor of conversion to AD in patients with MCI (Devanand et al., 2012). The *DMD* gene has been demonstrated to be associated with depressive disorder (Clark et al., 2012; Schosser et al., 2013; Blokland et al., 2022), educational attainment (Lee et al., 2018; Okbay et al., 2022), migraine (Hautakangas et al., 2022) and schizophrenia (Trubetskoy et al., 2022). SNP rs4596772, having a statistically significant effect on the mean values of the FreeSurfer middle temporal gyrus volume (i.e., FS MidTemp), is found near to the *TBX22* gene. One study demonstrated that the risk for the progression to the AD is reduced in the patients with the MCI having larger middle temporal gyrus volume (Desikan et al., 2009). It has been reported that the *TBX22* gene is significantly associated with autism spectrum disorder in Vietnamese children (Tran et al., 2020). SNP rs5929538 is found within the *LOC101928437* gene and has the significant effect on FDG PET, which can reflect the brain glucose metabolism mainly determined by synaptic activity (Hojjati and Babajani-Feremi, 2022). The glucose metabolism in the angular gyrus, temporal gyrus and posterior cingulate cortex has been reported to be significantly reduced in patients with the AD, compared to controls (Hunt et al., 2007). The *LOC101928437* gene is a novel candidate gene for non-syndromic intellectual disability in Han Chinese subjects of the Qinba region of China (Zhou et al., 2015). SNP rs2213488 is located in the *TENM1* gene and influences the volume of the hippocampus (i.e., FS Hippocampus). One study reported that the patients with the MCI had the increased rate of hippocampal volume loss when they converted to the AD (Moon et al., 2018). The *TENM1* gene is broadly expressed in brain, prostate and 17 other tissues, is related to the fear of minor pain (Randall et al., 2017), and may be associated with X-linked intellectual disability (Bengani et al., 2021). SNP rs5920524, located near to the *SPANXN1* gene, influences FDG PET. The *SPANXN1* gene has been demonstrated to be associated with brain shape (Naqvi et al., 2021). SNP rs5945306, within the *ZFP92* gene, has the significant effect on FAQ. The *ZFP92* gene exhibits predominant expression in pancreatic islets, with elevated levels also detected in the brain (Osipovich et al., 2023). Besides SNPs rs4829868 and rs5931111 with the *p*-values being less than $5\times 10^{-8}$ mentioned above, there were other seven SNPs (rs12157031, rs428303, rs5953487, rs10284107, rs5955016, rs6540385, and rs763320) identified in the longitudinal XWAS. SNPs rs6540385 and rs763320, which have the significant effects on the rate of the change of the FS Ventricles, are found located in the same gene, *AFF2*. One study showed that patients with the AD had the larger ventricular volume, compared to the MCI (Carmichael et al., 2007). CCG repeat expansions in the *AFF2* gene are associated with X-linked intellectual disability (Liu et al., 2021). Besides, the *AFF2* gene has been reported to be associated with autism spectrum disorder (Mondal et al., 2012), neuroticism (Luciano et al., 2021), and educational attainment (Okbay et al., 2022). SNPs rs12157031, rs428303, rs5953487, rs10284107, and rs5955016 were found to be located near to the *LOC124905257*, *NCKAP1P1*, *LOC105373344*, *RN7SKP149*, and *MTND1P33* genes, respectively. However, to the best of our knowledge, there have not been functional annotations for these five genes. These novel SNPs we discovered may be associated with the QBs of the AD, but this still requires to be confirmed by subsequent molecular genetics.

Note that most of the 16 QBs of the AD are statistically significantly correlated, with the *p*-values being less than $4.76\times 10^{-4}$ in ADNI cohort 1 (Supplementary Figure S5) and $4.17\times 10^{-4}$ in ADNI cohort GO/2 (Supplementary Figure S6), respectively. So, according to Carrion-Castillo et al. (2017), we did not consider further correcting the significance level based on these 16 QBs for multiple testing. On the other hand, the looser significance criterion for X chromosomes is generally used than autosomes. Therefore, in this article, we just set the corrected significance level of the association tests to be $0.05/19692=2.54\times 10^{-6}$ in ADNI cohorts 1, GO/2, and 1/GO/2, where 19,692 is the number of the X-chromosomal SNPs after the QC we used. In fact, even at the significance level of $0.05/\left(19692\times 16\right)=1.59\times 10^{-7}$ corrected based on these 16 QBs of the AD, we still identified three SNPs (rs5945306, rs4829868, and rs5931111). Specifically, SNP rs5945306 is statistically significantly associated with the transformed FAQ ( $p_{T_{\mathrm{chenw}}}=9.22\times 10^{-8}$ ) from Table 3. SNPs rs4829868 and rs5931111 are statistically significantly associated with the transformed FS Ventricles over time, with the respective *p*-values of the time×SNP interaction effects being $3.19\times 10^{-8}$ and $1.92\times 10^{-8}$ under the XCI-E from Table 5. Furthermore, we analyzed the ADNI database from both the cross-sectional and longitudinal perspectives, but unfortunately, the identified SNPs of these two perspectives could not be mutually validated (Supplementary Tables S8, S9, S11), and for the longitudinal XWAS, only the time×SNP interaction effects are statistically significant at the identified SNPs, while the SNP main effects are not (Supplementary Table S10). This may be due to the facts that the LMM needs to estimate more parameters and the models are more complicated, which require larger sample sizes than the cross-sectional XWAS to achieve the same statistical power (Uffelmann et al., 2021). Finally, we conducted two sensitive analyses as follows. Note that we identified 15 significant SNPs based on a cross-ethnic sample consisting of non-Hispanic White, non-Hispanic African American, Hispanic and others, by regarding the top 10 PCs of all the autosomal and X-chromosomal SNPs as the covariates included in the models to adjust the influence of the population stratification. To confirm that these significant SNP effects are not due to the population stratification, the first sensitive analysis was that we repeated the analysis just based on the non-Hispanic White subjects for these 15 SNPs, while not incorporating the top 10 PCs, like the studies (Li et al., 2018; Lee et al., 2022). The corresponding results were listed in Supplementary Tables S12–S14. There were 671, 701, and 1,384 non-Hispanic White subjects in ADNI cohorts 1, GO/2 and 1/GO/2, respectively (Table 2). In the cross-sectional XWAS, three SNPs (rs5927116, rs5920524, and rs5945306) among six retained their statistical significance (Supplementary Table S12). In the longitudinal XWAS, only the time×SNP interaction effects of two SNPs (rs6540385 and rs763320) among nine remained statistically significant (Supplementary Table S13). However, other 10 SNPs were not statistically significant, which may be due to the reduced sample size. On the other hand, it should be noted that the test statistics QXcat , ${\mathrm{QZ}}_{\max}$ , ${\mathrm{QMVX}}_{\mathrm{cat}}$ and ${\mathrm{QMVZ}}_{\max}$ for the cross-sectional XWAS are constructed by stratifying the data into females and males. However, in the longitudinal XWAS, we only included the sex as a covariate in the model to adjust the effect of the sex. To investigate what gender drives the significant time×SNP interaction effects of the nine SNPs in the longitudinal XWAS, the second sensitive analysis was that we additionally performed the longitudinal XWAS in females and males separately for these nine SNPs. Unfortunately, there was no significant time×SNP interaction effect to be found, probably because the sample size for each gender was reduced when conducting the longitudinal XWAS stratified by the sex (the results omitted for brevity).

In this article, we considered the following issues. (1) Facing the challenge that the 11 QBs do not follow normal distributions, we used the rank-based inverse normal transformation method on these 11 QBs to avoid increasing false positive results; (2) We analyzed the ADNI database from both the cross-sectional and longitudinal perspectives, which allow us to make full use of the ADNI data; (3) 15 X-chromosomal SNPs for the AD were identified by taking into full consideration the XCI patterns. On the other hand, there are several limitations in this article which need to be discussed. Firstly, the onset of the AD is closely related to a variety of genetic and non-genetic factors. However, in addition to genetic information, this article only included a few covariates (sex, age, educational level, and *APOE4* allelic dosage), which may affect the accuracy of the results to a certain extent. Secondly, our work may be only an exploratory study and provide a reference for follow-up researches since the sample size is not so large.

## Data availability statement

Publicly available datasets were analyzed in this study. This data can be found at: https://adni.loni.usc.edu/about.

## Ethics statement

Ethical approval was not required for the study involving humans in accordance with the local legislation and institutional requirements. Written informed consent to participate in this study was not required from the participants or the participants' legal guardians/next of kin in accordance with the national legislation and the institutional requirements.

## Author contributions

K-WW: Conceptualization, Formal analysis, Methodology, Writing – original draft. Y-XY: Conceptualization, Formal analysis, Methodology, Writing – original draft. BZ: Conceptualization, Formal analysis, Methodology, Writing – original draft. YZ: Writing – review & editing, Software, Validation. Y-FW: Writing – review & editing, Visualization. F-SM: Writing – review & editing, Visualization. SZ: Writing – review & editing, Validation. J-XW: Writing – review & editing, Validation. J-YZ: Writing – review & editing, Conceptualization, Project administration, Supervision.

## Footnotes

^1^https://adni.loni.usc.edu/about/

^2^http://www.pi4cs.org/qt-pad-challenge

^3^https://gtexportal.org/home/

^4^https://genome.ucsc.edu/

^5^https://www.ncbi.nlm.nih.gov/

^6^https://www.genecards.org/
