# Supplementary material for: X chromosome-wide association study of quantitative biomarkers from the Alzheimer’s Disease Neuroimaging Initiative study
Source: Front Aging Neurosci. 2023 Nov 14;15:1277731. doi: 10.3389/fnagi.2023.1277731 (PMC10682795; doi:10.3389/fnagi.2023.1277731)
Supplement: Supplementary file 1 [file Presentation_1.pdf]

Supplementary Material

X chromosome-wide association study of quantitative biomarkers from the Alzheimer's Disease Neuroimaging Initiative Study

Kai-Wen Wang†, Yu-Xin Yuan†, Bin Zhu†, Yi Zhang, Yi-Fang Wei, Fan-Shuo Meng, Shun Zhang, Jing-Xuan Wang and Ji-Yuan Zhou* for the Alzheimer's Disease Neuroimaging Initiative‡

*** Correspondence:** Corresponding Author: zhoujiyuan5460@hotmail.com

†These authors have contributed equally to this work and share first authorship

‡Data used in preparation of this article were obtained from the Alzheimer's Disease Neuroimaging Initiative (ADNI) database (https://adni.loni.usc.edu/about/). As such, the investigators within the ADNI contributed to the design and implementation of ADNI and/or provided data but did not participate in analysis or writing of this report. A complete listing of ADNI investigators can be found at: http://adni.loni.usc.edu/wp-content/uploads/how_to_apply/ADNI_Acknowledgement_List.pdf

1. Supplementary methods. Overview of cross-sectional association analysis methods for X chromosome

In this article, we used the methods testing for means (i.e., QXcat, $\mathrm{QZ_{max}}$, $\mathrm{T_{chenw}}$, and $\mathrm{T_{plinkw}}$), the method testing for variances (i.e., wM3VNA3.3), and the methods simultaneously testing for means and variances (i.e., $\mathrm{QMVX_{cat}}$ and $\mathrm{QMVZ_{max}}$) (Yang et al., 2022) to conduct the cross-sectional XWAS. The overview of these methods are as follows.

Consider an SNP of interest on the X chromosome with alleles a and A, where A is the minor allele. Suppose that we collect a sample of N independent subjects consisting of N_f females and N_m males. Let $\mathbf{Y} = (y_1, y_2, \dots, y_N)^T$ denote a vector of the values of the original or transformed QB under study at the baseline for all the N subjects, and $\mathbf{Y}_f = (y_{f1}, y_{f2}, \dots, y_{fN_f})^T$ and $\mathbf{Y}_m = (y_{m1}, y_{m2}, \dots, y_{mN_m})^T$ be the subvectors of $\mathbf{Y}$ for N_f females and N_m males, respectively. Let $\mathbf{G} = (G_1, G_2, \dots, G_N)^T$ denote a vector of the numbers of allele A in the genotypes of all the N subjects, and $\mathbf{G}_f = (G_{f1}, G_{f2}, \dots, G_{fN_f})^T$ and $\mathbf{G}_m = (G_{m1}, G_{m2}, \dots, G_{mN_m})^T$ be the subvectors of $\mathbf{G}$ for N_f females and N_m males, respectively. For females, G_{fi} takes the values of 0, 1, and 2 for genotypes aa, Aa, and AA, respectively; for males, G_{mi} takes the values of 0 and 1 for genotypes a and A, respectively. In females, the means of the original or transformed QB for genotypes aa, Aa, and AA are represented by μ_{f0}, μ_{f1}, and μ_{f2}, respectively, and the variances of the original or transformed QB for genotypes aa, Aa, and AA are denoted as σ_{f0}^2, σ_{f1}^2, and σ_{f2}^2, respectively. In males, the means of the original or transformed QB for genotypes a and A are represented by μ_{m0} and μ_{m1}, respectively, and the variances of the original or transformed QB for genotypes a and A are denoted as σ_{m0}^2 and σ_{m1}^2, respectively. Here, we consider three types of null hypotheses of no association between the original or transformed QB and the SNP. $\mathrm{H_0^M}$: only the means of the original or transformed QB across genotypes are equal (i.e., $\mu_{f0} = \mu_{f1} = \mu_{f2}, \mu_{m0} = \mu_{m1}$ and no restrictions on the variances), $\mathrm{H_0^V}$: only

the variances of the original or transformed QB across genotypes are equal (i.e., $\sigma_{f0}^2 = \sigma_{f1}^2 = \sigma_{f2}^2, \sigma_{m0}^2 = \sigma_{m1}^2$ and no restrictions on the means) and $\mathrm{H}_0^{\mathrm{MV}}$: both the means and the variances of the original or transformed QB across genotypes are equal (i.e., $\mu_{f0} = \mu_{f1} = \mu_{f2}$, $\mu_{m0} = \mu_{m1}$, $\sigma_{f0}^2 = \sigma_{f1}^2 = \sigma_{f2}^2$ and $\sigma_{m0}^2 = \sigma_{m1}^2$).

1.1. Methods testing for means

QXcat and $\mathrm{QZ}_{\max}$ are two sex-stratified X-chromosomal association tests for the means of the original or transformed QB. For females, the model can be written as

$$y_{fi} = \beta_{f0} + \beta_{f1}X_{fi}^{(1)} + \beta_{f2}X_{fi}^{(2)} + \boldsymbol{b}_f^T\mathbf{Z}_{fi} + \varepsilon_{fi}, i = 1, 2, \dots, N_f, \tag{S1}$$

where β_{f0} is the intercept; $X_{fi}^{(1)} = I_{\{G_{fi}\geq 1\}}$ and $X_{fi}^{(2)} = I_{\{G_{fi}=2\}}$ are two indicator variables with respect to G_{fi}, and the corresponding regression coefficients respectively are β_{f1} and β_{f2}; $\mathbf{Z}_{fi}$ denotes a vector of the covariates (i.e., age, education level, *APOE4* allelic dosage, the top 10 principal components, and the batch) for female i with the corresponding regression coefficients being $\boldsymbol{b}_f$; ε_{fi} is a random error which follows $N(0, \sigma_{f0}^2)$, $N(0, \sigma_{f1}^2)$ and $N(0, \sigma_{f2}^2)$ for genotypes aa, Aa and AA, respectively. For males, we use the following model to test for the association between the original or transformed QB and the SNP

$$y_{mi} = \beta_{m0} + \beta_{m1}G_{mi} + \boldsymbol{b}_m^T\mathbf{Z}_{mi} + \varepsilon_{mi}, i = 1, 2, \dots, N_m, \tag{S2}$$

where β_{m0} is the intercept and β_{m1} is the regression coefficient of G_{mi}; $\mathbf{Z}_{mi}$ is a vector of the covariates (i.e., age, education level, *APOE4* allelic dosage, the top 10 principal components, and the batch) for male i with the corresponding regression coefficients being $\boldsymbol{b}_m$; ε_{mi} is a random error which follows $N(0, \sigma_{m0}^2)$ and $N(0, \sigma_{m1}^2)$ for genotypes a and A, respectively. Since some factors (such as genotype-by-environment interactions (Wang et al., 2019a) and XCI (Deng et al., 2019)) may lead to unequal variances of the original or transformed QB across different genotypes, the parameters of models (S1) and (S2) can be estimated by the weighted least square method. Through jointly testing $\mathrm{H}_0^{\mathrm{M}}$: $\beta_{f1} = \beta_{f2} = \beta_{m1} = 0$, we can get the p-values of QXcat and $\mathrm{QZ}_{\max}$ by considering various XCI patterns (Wang et al., 2014; Yu et al., 2022) and different dosage compensation patterns (Wang et al., 2019b), respectively. The main difference between QXcat and $\mathrm{QZ}_{\max}$ is the computing process of the p-value and the details please refer to Yang et al. (2022).

For $\mathrm{T_{plinkw}}$, which is the weighted version of $\mathrm{T_{plink}}$ (Özbek et al., 2018), the model can be written as

$$y_i = \alpha_0 + \alpha_S S_i + \alpha_A G_i + \alpha_{GS} G_i S_i + \boldsymbol{\varphi}^T \boldsymbol{Z}_i + \varepsilon_i, i = 1, 2, \dots, N, \tag{S3}$$

where α_0 is the intercept; S_i is the sex of subject i with the regression coefficient being α_S, where females are coded as 0 and males are coded as 1; α_A and α_{GS} are the regression coefficients of G_i and the interaction term $G_i S_i$, respectively; $\boldsymbol{Z}_i$ is a vector of the covariates without sex (i.e., age, education level, *APOE4* allelic dosage, the top 10 principal components, and the batch) for subject i with the corresponding regression coefficients being $\boldsymbol{\varphi}$; ε_i is a random error. Then, testing for the differences of the means of the original or transformed QB is achieved by testing $\mathrm{H}_0^{\mathrm{M}}$: $\alpha_A = \alpha_{GS} = 0$ via the standard regression F test, where the model is fitted using the weighted least square method. Finally, $\mathrm{T_{chenw}}$ is obtained by adding a variable indicative of heterozygous females (Chen et al., 2021) in model (S3), i.e.,

$$y_i = \xi_0 + \xi_S S_i + \xi_A G_i + \xi_D G_{Di} + \xi_{GS} G_i S_i + \boldsymbol{\theta}^T \boldsymbol{Z}_i + \varepsilon_i, i = 1, 2, \ldots, N, \quad \text{(S4)}$$

where $G_{Di} = I_{\{G_i=1 \text{ and } S_i=0\}}$ is indicative of whether or not subject i being a heterozygous female, and ξ_D is its regression coefficient. Through jointly testing $\mathrm{H_0^M}$: $\xi_A = \xi_D = \xi_{GS} = 0$, we can check whether the SNP has the effects on the mean values of the original or transformed QB.

1.2. Methods testing for variances

wM3VNA3.3 is a two-stage method to test the effect of the SNP on phenotypic variances (Deng et al., 2019). In stage 1, the original or transformed QB is adjusted for a shift in location (mean or median) using the model below

$$y_i = \eta_0 + \eta_S S_i + \eta_A G_i + \eta_{GS} G_i S_i + \boldsymbol{\vartheta}^T \boldsymbol{Z}_i + r_i, i = 1, 2, \ldots, N, \quad \text{(S5)}$$

where the estimated residuals (denoted by $\hat{r}_i$'s) can be calculated via the least absolute deviations method. In stage 2, the response ($d_i = |\hat{r}_i|$) is the absolute residual obtained from stage 1. Then, the model can be written as

$$d_i = \delta_0 + \delta_{G1} G_i^{(1)} + \delta_{G2} G_i^{(2)} + \delta_S S_i + \delta_{G1S} G_i^{(1)} S_i + e_i, i = 1, 2, \ldots, N, \quad \text{(S6)}$$

where $G_i^{(1)} = I_{\{G_i=1\}}$ and $G_i^{(2)} = I_{\{G_i=2\}}$ are two indicator variables, respectively, for the $Aa + A$ and AA groups, and δ_{G1} and δ_{G2} are the corresponding regression coefficients; δ_{G1S} is the regression coefficient of the interaction term $G_i^{(1)} S_i$, and e_i is a random error. As such, testing for variance heterogeneity is achieved by testing $\mathrm{H_0^V}$: $\delta_{G1} = \delta_{G2} = \delta_{G1S} = 0$ via the standard regression F test, where the model is fitted using the ordinary least square method.

1.3. Methods simultaneously testing for means and variances

When both the means and the variances of the original or transformed QB across different genotypes are different, a more powerful approach to identify the susceptibility loci is to jointly test for the mean and variance effects. Based on this, Yang et al. (2022) further proposed two mean-variance-based tests $\mathrm{QMVX_{cat}}$, by combining $p_{\mathrm{wM3VNA3.3}}$ with p_{QXcat}, and $\mathrm{QMVZ_{max}}$, by combining $p_{\mathrm{wM3VNA3.3}}$ with $p_{\mathrm{QZ_{max}}}$, based on Fisher's method (Fisher et al., 1967), i.e.,

$$\mathrm{QMVX_{cat}} = -2\ln(p_{\mathrm{QXcat}} p_{\mathrm{wM3VNA3.3}})$$

and

$$\mathrm{QMVZ_{max}} = -2\ln(p_{\mathrm{QZ_{max}}} p_{\mathrm{wM3VNA3.3}}).$$

Under $\mathrm{H_0^{MV}}$, both $\mathrm{QMVX_{cat}}$ and $\mathrm{QMVZ_{max}}$ asymptotically follow a chi-square distribution with the degrees of freedom being 4 (Chen et al., 2017).

2. Supplementary results. Inferring the direction of the SNP effect on the QB in cross-sectional XWAS

After identifying the statistically significantly associated SNPs in the cross-sectional XWAS, another important issue is to check the direction of the SNP effect on the original or transformed QB. However, due to the complexity of the cross-sectional XWAS caused by taking account of various XCI or dosage compensation patterns, it seems difficult to directly determine the effect size and the effect direction. Fortunately, by observing the signs of the regression coefficients in the mean-based tests (QXcat and QZ_{max}) and those in the variance-based test (wM3VNA3.3), we can infer the direction of the SNP effect on the means and the variances, respectively. Specifically, for the mean-based tests (QXcat and QZ_{max}), we can examine the signs of the estimates $\hat{\beta}_{f1}$, $\hat{\beta}_{f2}$ and $\hat{\beta}_{m1}$ of the regression coefficients β_{f1}, β_{f2} and β_{m1}, as presented in models (S1) and (S2) of Supplementary Methods. In females, β_{f1} can be regarded as the mean effect of genotype Aa relative to genotype aa, and the mean effect of genotype AA compared to aa can be denoted by $\beta_{f1} + \beta_{f2}$. In males, β_{m1} can be treated as the mean effect of genotype A relative to genotype a. For the other two mean-based tests (T_{chenw} and T_{plinkw}), we can also check the direction of the SNP effect on the means of the original or transformed QB by respectively observing the signs of the regression coefficients in models (S4) and (S3) of Supplementary Methods, and the drawn conclusions are almost consistent with those from QXcat and QZ_{max}. So, we will not repeat them here for brevity. On the other hand, for the variance-based test (wM3VNA3.3), we can get the estimates $\hat{\delta}_{G1}$, $\hat{\delta}_{G2}$ and $\hat{\delta}_{G1S}$ of the regression coefficients δ_{G1}, δ_{G2} and δ_{G1S} in stage 2, as shown in model (S6) of Supplementary Methods. δ_{G1} and δ_{G2} represent the effects of genotypes Aa and AA on the variances of the original or transformed QB compared to aa in females, respectively, and $\delta_{G1} + \delta_{G1S}$ can be regarded as the effect of genotype A on the variances of the original or transformed QB relative to genotype a in males.

Supplementary Table S6 displays the point estimates and the 95% confidence intervals (CIs) of the regression coefficients β_{f1}, β_{f2}, and β_{m1} in QXcat and QZ_{max} for six statistically significantly associated SNPs found in the cross-sectional XWAS, and Supplementary Table S7 shows the point estimates and the 95% CIs of the regression coefficients δ_{G1}, δ_{G2}, and δ_{G1S} in stage 2 of wM3VNA3.3 for these SNPs. SNP rs5927116 is in the *DMD* gene, which has the effects on the mean values of the FS Entorhinal ($p_{\text{QXcat}} = 1.74 \times 10^{-6}$). Note that $\hat{\beta}_{f1} = 383.656$ and $\hat{\beta}_{m1} = 103.977$ in QXcat and QZ_{max} are bigger than 0, with the corresponding CIs being 202.911 ~ 564.401 and 28.151 ~ 179.804, respectively. So, for SNP rs5927116, the mean FS Entorhinal for genotype TC is higher than that for genotype CC in females and the mean FS Entorhinal for genotype T is larger than that for genotype C in males. Meanwhile, $\hat{\beta}_{f2} = 20.785$ and the 95% CI is -139.401 ~ 180.971, while $\hat{\beta}_{f1} + \hat{\beta}_{f2}$ is still higher than 0. As such, the mean FS Entorhinal for genotype TT is larger than that for genotype CC and is not significantly different from that for genotype TC in females. This means that the minor allele T at rs5927116 is a causal allele, which will increase the mean FS Entorhinal. SNP rs4596772 only influences the mean values of the FS MidTemp ($p_{\text{QXcat}} = 9.94 \times 10^{-7}$ and $p_{\text{QZ}_{\text{max}}} = 7.55 \times 10^{-7}$). The point estimates (95% CIs) of the regression coefficients β_{f1}, β_{f2}, and β_{m1} in QXcat and QZ_{max} are -1877.788 (-2806.175 ~ -949.400), -259.028 (-1006.061 ~ 488.004) and -464.309 (-790.651 ~ -137.966), respectively, suggesting that the direction of its effect on the FS MidTemp is just opposite to that of SNP rs5927116 on the FS Entorhinal. Thus, the minor allele A at rs4596772 is a causal allele, which will decrease the mean FS MidTemp. SNP rs5929538 is included in the *LOC101928437* gene and has the effects on the mean values of the transformed FDG PET ($p_{\text{T}_{\text{chenw}}} = 2.28 \times 10^{-6}$). Note that $\hat{\beta}_{f1} = 0.223$ (95% CI: -0.200 ~ 0.646) and $\hat{\beta}_{f2} = -1.175 < 0$ (95% CI: -1.616 ~ -0.735) in

QXcat and QZ_{max}, so females with genotype AA at rs5929538 have less FDG PET than those with genotype GG, while the mean effect of genotype AG compared to genotype GG in females (because the 95% CI of β_{f1} contains 0) and the mean effect of genotype A relative to genotype G in males ($\hat{\beta}_{m1} = 0.103$; 95% CI: -0.032 ~ 0.239) are not statistically significant. Moreover, for SNP rs2213488, which is located in the *TENM1* gene, only the p-values of $QMVX_{cat}$ and $QMVZ_{max}$ for simultaneously testing for the means and the variances of the FS Hippocampus are lower than the significance level 2.54×10^{-6}, where the corresponding p-values are 1.30×10^{-6} and 7.23×10^{-7}, respectively. This suggests that either the means or the variances of the FS Hippocampus across different genotypes are different, which needs to be further investigated for larger sample size. SNP rs5920524 has the effects on the mean values of the transformed FDG PET ($p_{\mathrm{QXcat}} = 5.57 \times 10^{-7}$ and $p_{\mathrm{T_{chenw}}} = 5.97 \times 10^{-7}$), and the resulting p-value of $QMVX_{cat}$ is 1.72×10^{-6}. Note that the point estimate (95% CI) of β_{f1} at rs5920524 is -0.074 (-0.346 ~ 0.199), so the mean effect of genotype TC compared to genotype CC in females is not statistically significant. In addition, both $\hat{\beta}_{f2} = 0.465$ and $\hat{\beta}_{m1} = 0.198$ in QXcat and QZ_{max} are larger than 0 and the corresponding CIs 0.223 ~ 0.707 and 0.101 ~ 0.295 do not contain 0, so females with genotype TT at rs5920524 tend to have greater mean of the transformed FDG PET compared to females with genotype CC, and the mean value of the transformed FDG PET for genotype T is greater than that for genotype C in males. This implies that the major allele C at rs5920524 is a risk allele and will decrease the FDG PET. Finally, SNP rs5945306 only influences the mean values of the transformed FAQ ($p_{\mathrm{QXcat}} = 7.67 \times 10^{-7}$ and $p_{\mathrm{T_{chenw}}} = 9.22 \times 10^{-8}$), and the resulting p-value of $QMVX_{cat}$ is 1.82×10^{-6}. Meanwhile, the point estimates (95% CIs) of the regression coefficients β_{f1}, β_{f2}, and β_{m1} in QXcat and QZ_{max} are -0.639 (-0.899 ~ -0.380), -0.053 (-0.216 ~ 0.109) and -0.004 (-0.093 ~ 0.084), respectively, indicating that the mean values of the transformed FAQ for genotypes CC and CT are lower than that for genotype TT in females, and the mean effect of genotype C compared to genotype T in males is not statistically significant. Therefore, the major allele T at rs5945306 in females is a risk allele and tends to increase the FAQ.

3. Supplementary Figures and Tables

3.1. Supplementary Figures

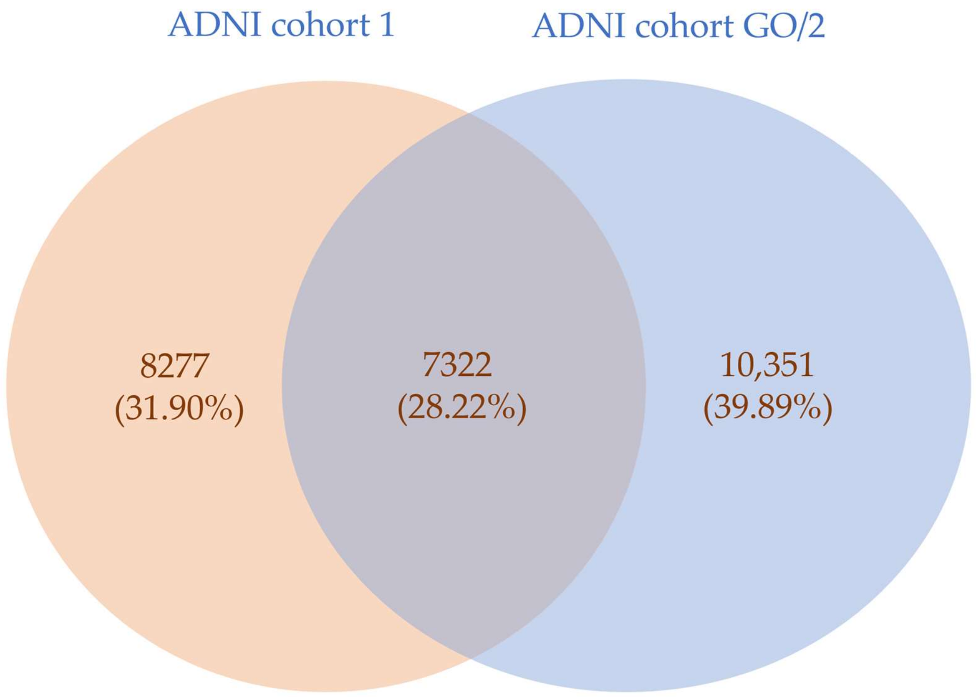

Supplementary Figure S1. Venn diagram of X-chromosomal SNPs in ADNI cohorts 1 and GO/2 before quality control.

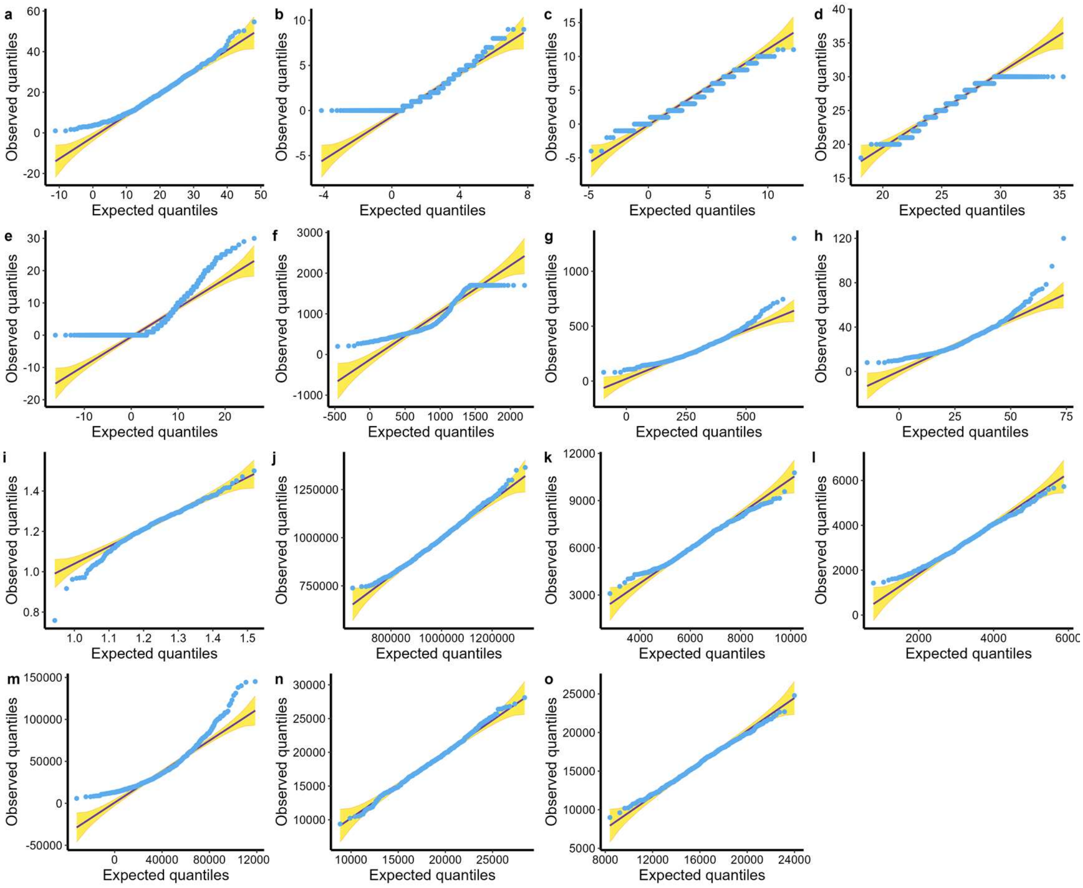

Supplementary Figure S2. Q-Q plots for 15 QBs at the baseline in ADNI cohort 1 ($N = 741$). (**a**) ADAS13; (**b**) CDRSB; (**c**) RAVLT.learning; (**d**) MMSE; (**e**) FAQ; (**f**) CSF ABETA; (**g**) CSF TAU; (**h**) CSF PTAU; (**i**) FDG PET; (**j**) FS WholeBrain; (**k**) FS Hippocampus; (**l**) FS Entorhinal; (**m**) FS Ventricles; (**n**) FS MidTemp; (**o**) FS Fusiform.

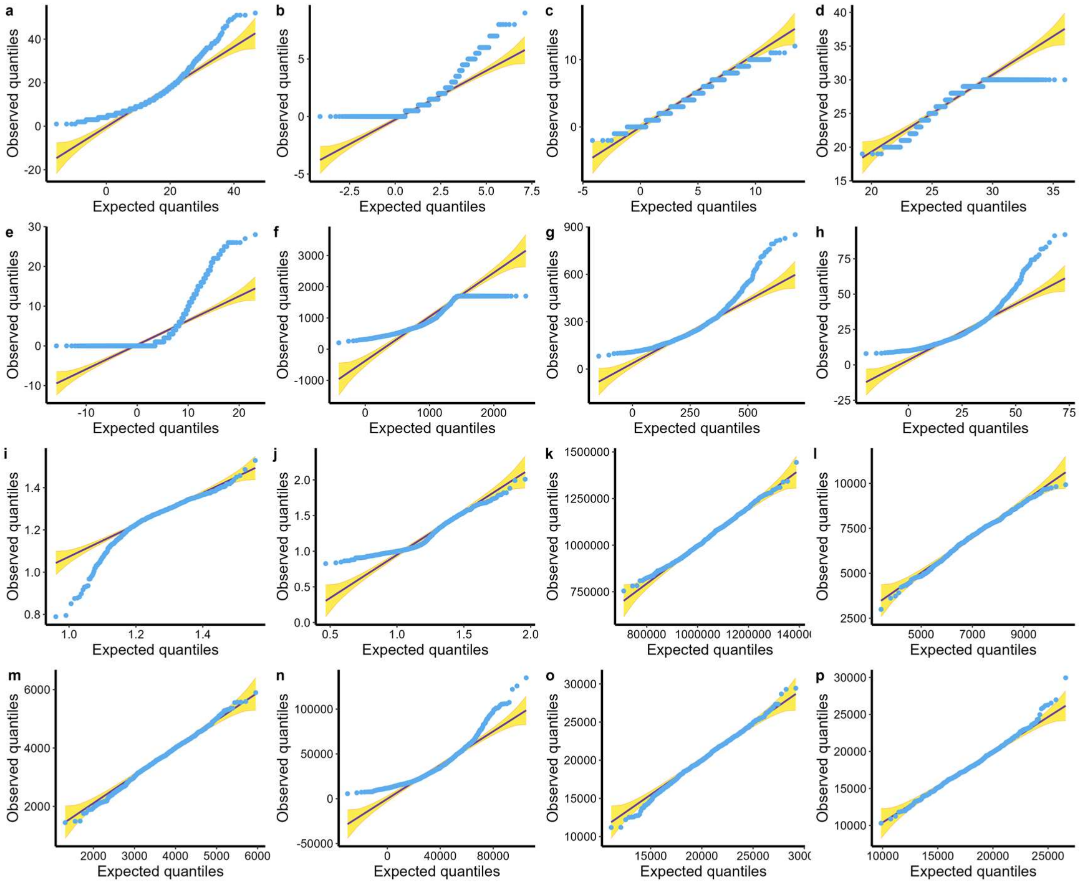

Supplementary Figure S3. Q-Q plots for 16 QBs at the baseline in ADNI cohort GO/2 ($N = 792$). (**a**) ADAS13; (**b**) CDRSB; (**c**) RAVLT.learning; (**d**) MMSE; (**e**) FAQ; (**f**) CSF ABETA; (**g**) CSF TAU; (**h**) CSF PTAU; (**i**) FDG PET; (**j**) Amyloid PET/AV45; (**k**) FS WholeBrain; (**l**) FS Hippocampus; (**m**) FS Entorhinal; (**n**) FS Ventricles; (**o**) FS MidTemp; (**p**) FS Fusiform.

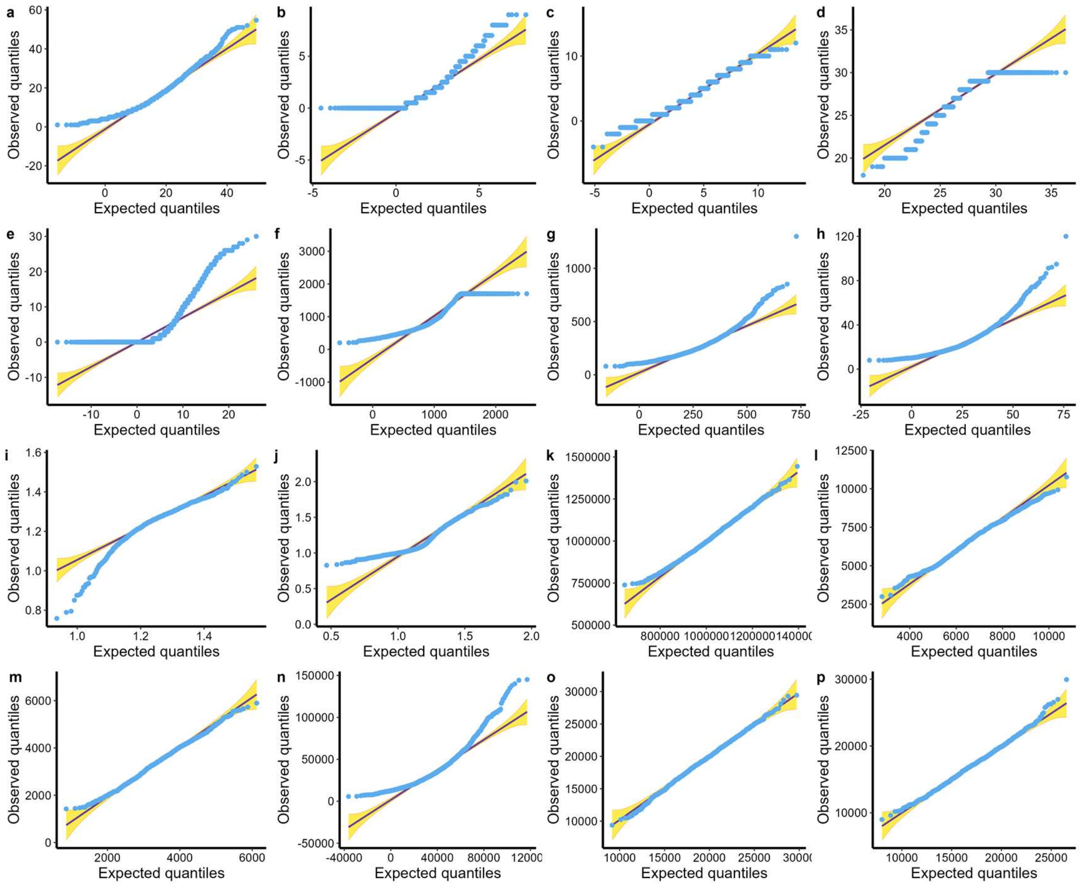

Supplementary Figure S4. Q-Q plots for 16 QBs at the baseline in ADNI cohort 1/GO/2 ($N = 1{,}546$). (**a**) ADAS13; (**b**) CDRSB; (**c**) RAVLT.learning; (**d**) MMSE; (**e**) FAQ; (**f**) CSF ABETA; (**g**) CSF TAU; (**h**) CSF PTAU; (**i**) FDG PET; (**j**) Amyloid PET/AV45; (**k**) FS WholeBrain; (**l**) FS Hippocampus; (**m**) FS Entorhinal; (**n**) FS Ventricles; (**o**) FS MidTemp; (**p**) FS Fusiform.

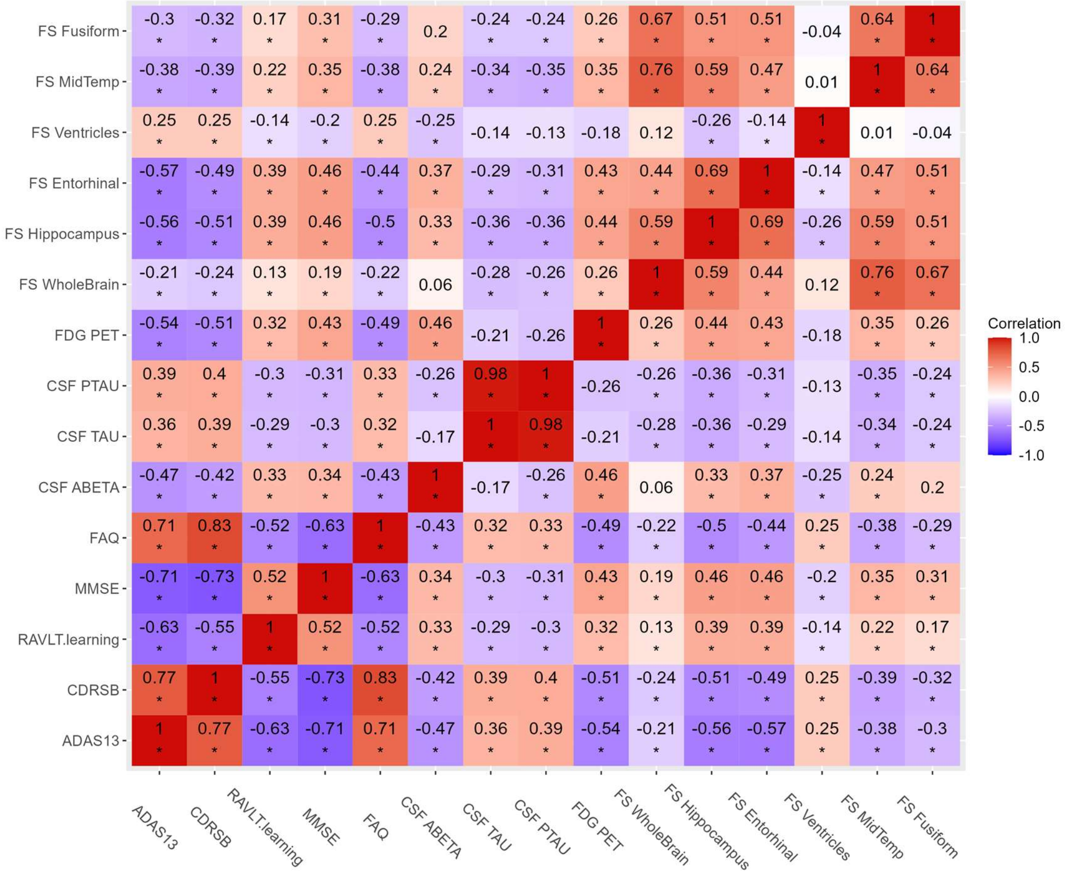

Supplementary Figure S5. Correlation analysis for 15 QBs at the baseline in ADNI cohort 1 ($N = 741$). The Pearson's correlation coefficients are computed among FS WholeBrain, FS Hippocampus, FS Entorhinal, FS MidTemp, and FS Fusiform, since they asymptotically follow the normal distributions. All other possible pairs of the QBs are analyzed with the Spearman's rank correlation coefficients. Correlations which are statistically significant after Bonferroni correction (p-value $< \frac{0.05}{105} = 4.76 \times 10^{-4}$, where $105 = 15 \times 14/2$) are marked with an asterisk.

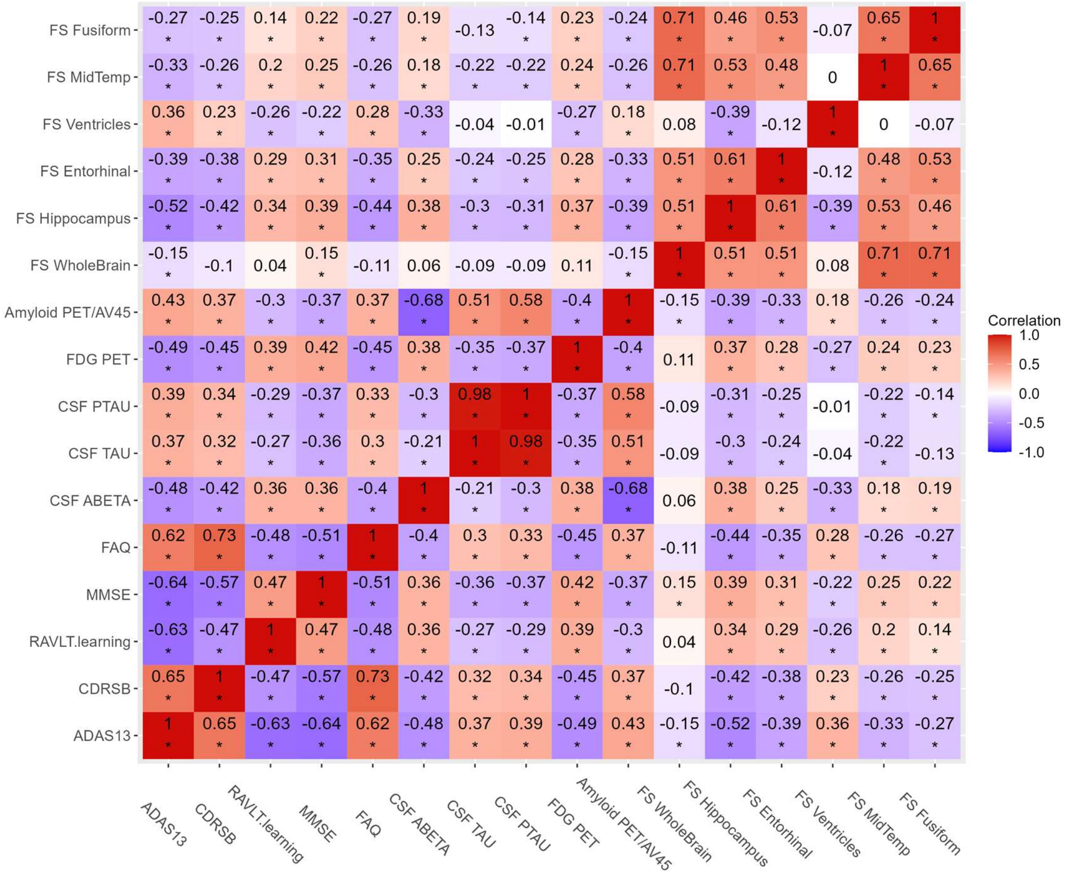

Supplementary Figure S6. Correlation analysis for 16 QBs at the baseline in ADNI cohort GO/2 ($N = 792$). The Pearson's correlation coefficients are computed among FS WholeBrain, FS Hippocampus, FS Entorhinal, FS MidTemp, and FS Fusiform, since they asymptotically follow the normal distributions. All other possible pairs of the QBs are analyzed with the Spearman's rank correlation coefficients. Correlations which are statistically significant after Bonferroni correction (p-value $< \frac{0.05}{120} = 4.17 \times 10^{-4}$, where $120 = 16 \times 15/2$) are marked with an asterisk.

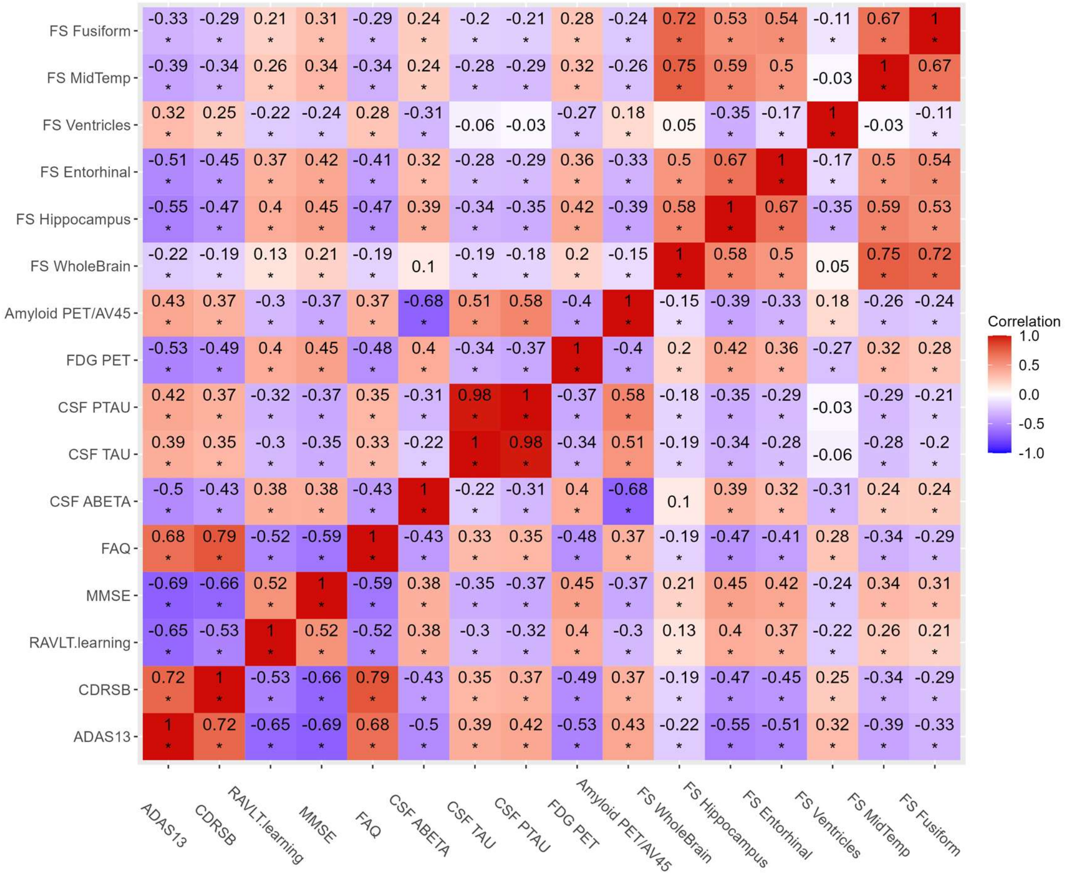

Supplementary Figure S7. Correlation analysis for 16 QBs at the baseline in ADNI cohort 1/GO/2 ($N = 1{,}546$). The Pearson's correlation coefficients are computed among FS WholeBrain, FS Hippocampus, FS Entorhinal, FS MidTemp, and FS Fusiform, since they asymptotically follow the normal distributions. All other possible pairs of the QBs are analyzed with the Spearman's rank correlation coefficients. Correlations which are statistically significant after Bonferroni correction (p-value $< \frac{0.05}{120} = 4.17 \times 10^{-4}$, where $120 = 16 \times 15/2$) are marked with an asterisk.

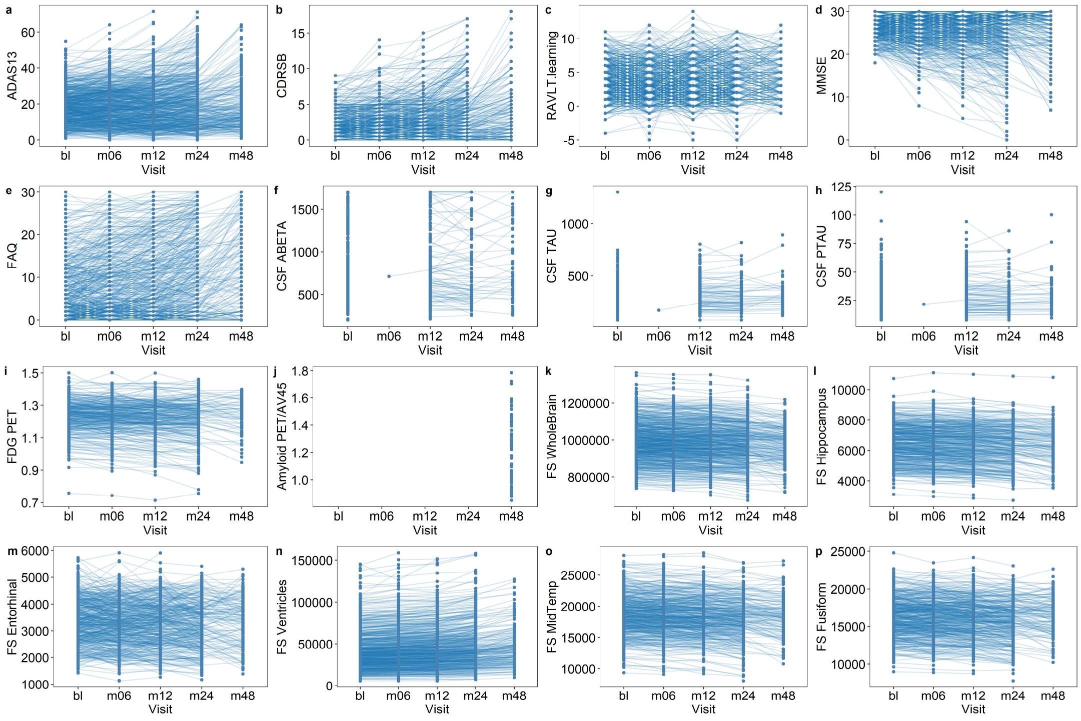

Supplementary Figure S8. Spaghetti plots of longitudinal course of 16 QBs in ADNI cohort 1 ($N = 741$). (**a**) ADAS13; (**b**) CDRSB; (**c**) RAVLT.learning; (**d**) MMSE; (**e**) FAQ; (**f**) CSF ABETA; (**g**) CSF TAU; (**h**) CSF PTAU; (**i**) FDG PET; (**j**) Amyloid PET/AV45; (**k**) FS WholeBrain; (**l**) FS Hippocampus; (**m**) FS Entorhinal; (**n**) FS Ventricles; (**o**) FS MidTemp; (**p**) FS Fusiform.

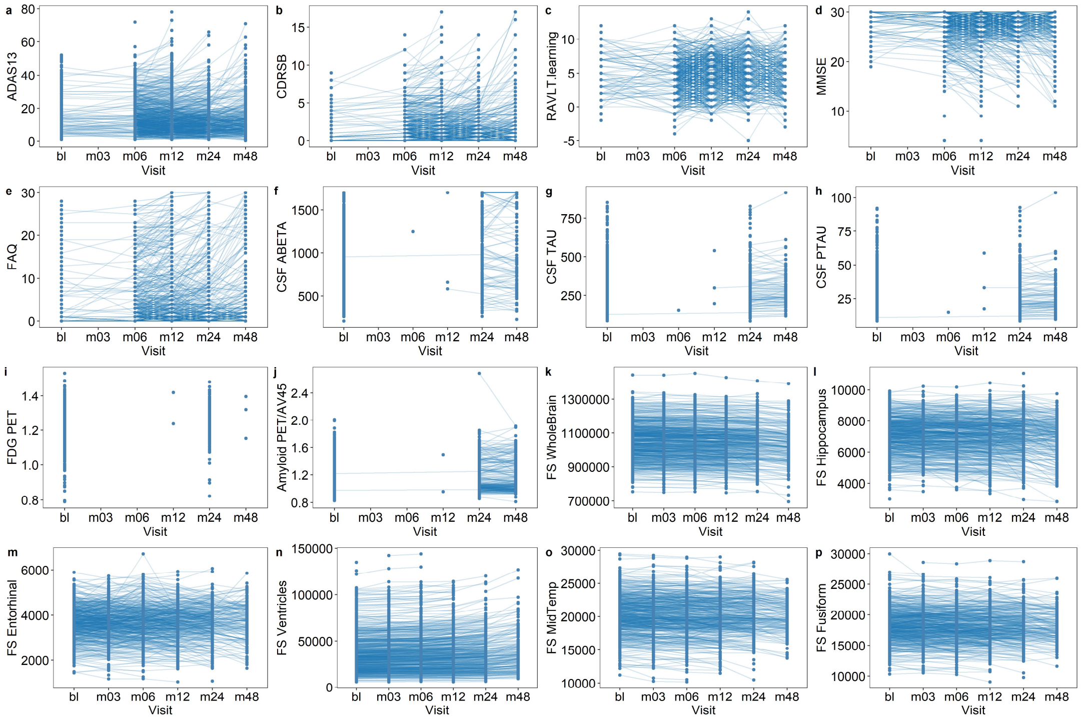

Supplementary Figure S9. Spaghetti plots of longitudinal course of 16 QBs in ADNI cohort GO/2 ($N = 792$). (**a**) ADAS13; (**b**) CDRSB; (**c**) RAVLT.learning; (**d**) MMSE; (**e**) FAQ; (**f**) CSF ABETA; (**g**) CSF TAU; (**h**) CSF PTAU; (**i**) FDG PET; (**j**) Amyloid PET/AV45; (**k**) FS WholeBrain; (**l**) FS Hippocampus; (**m**) FS Entorhinal; (**n**) FS Ventricles; (**o**) FS MidTemp; (**p**) FS Fusiform.

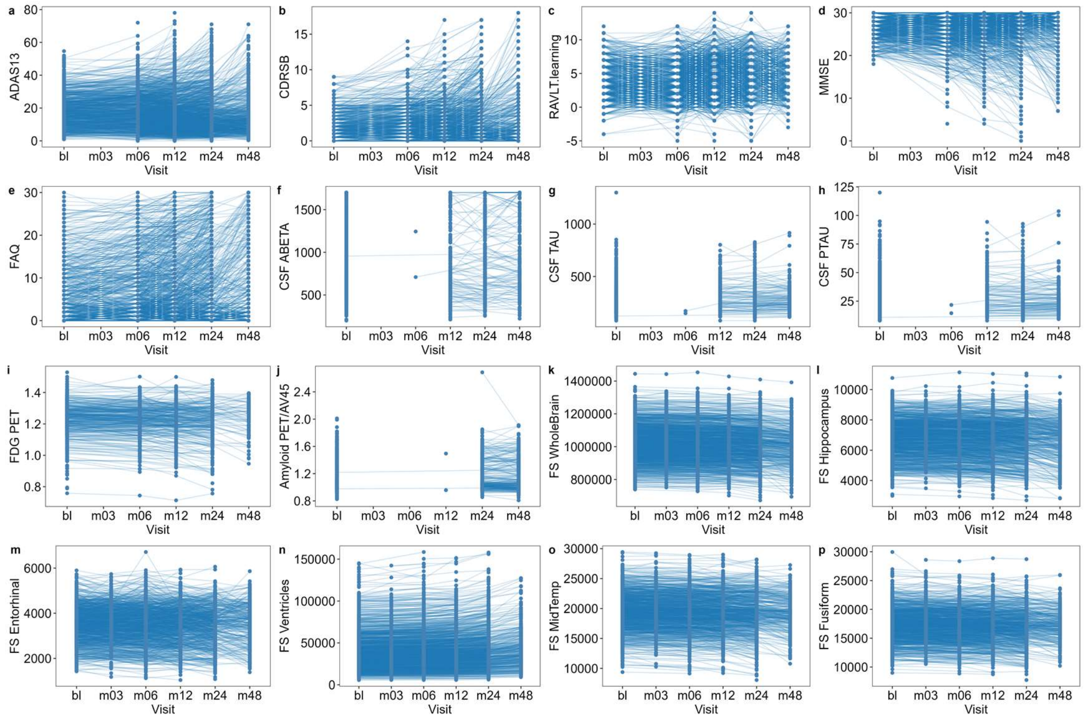

Supplementary Figure S10. Spaghetti plots of longitudinal course of 16 QBs in ADNI cohort 1/GO/2 ($N = 1{,}546$). (**a**) ADAS13; (**b**) CDRSB; (**c**) RAVLT.learning; (**d**) MMSE; (**e**) FAQ; (**f**) CSF ABETA; (**g**) CSF TAU; (**h**) CSF PTAU; (**i**) FDG PET; (**j**) Amyloid PET/AV45; (**k**) FS WholeBrain; (**l**) FS Hippocampus; (**m**) FS Entorhinal; (**n**) FS Ventricles; (**o**) FS MidTemp; (**p**) FS Fusiform.

3.2. Supplementary Tables

Supplementary Table S1. Sample size and number of used SNPs in ADNI cohorts 1, GO/2, and 1/GO/2 for cross-sectional XWAS.

Category	Trait	ADNI cohort 1		ADNI cohort GO/2		ADNI cohort 1/GO/2	
		N[a]	N_{snp}[b]	N[a]	N_{snp}[b]	N[a]	N_{snp}[b]
Cognition	ADAS13	733	6,781	788	7,731	1,534	4,924
Cognition	CDRSB	741	6,793	792	7,782	1,546	4,942
Cognition	RAVLT.learning	738	6,784	791	7,779	1,541	4,938
Cognition	MMSE	741	6,793	792	7,782	1,546	4,942
Cognition	FAQ	738	6,741	790	7,777	1,541	4,940
CSF	CSF ABETA	364	3,538	718	7,347	1,089	4,325
CSF	CSF TAU	364	3,538	718	7,347	1,089	4,325
CSF	CSF PTAU	364	3,538	718	7,347	1,089	4,325
PET	FDG PET	366	2,830	783	7,739	1,154	4,471
PET	Amyloid PET/AV45[c]	-	-	777	7,710	777	3,811
MRI	FS WholeBrain	731	6,781	760	7,638	1,504	4,901
MRI	FS Hippocampus	606	6,018	722	7,484	1,338	4,739
MRI	FS Entorhinal	606	6,018	687	7,400	1,303	4,715
MRI	FS Ventricles	728	6,772	737	7,528	1,478	4,887
MRI	FS MidTemp	606	6,018	687	7,400	1,303	4,715
MRI	FS Fusiform	606	6,018	687	7,400	1,303	4,715

[a]N: sample size.
[b]N_{snp}: the number of the SNPs in ADNI cohorts 1, GO/2 or 1/GO/2.
[c]The values of Amyloid PET/AV45 at baseline are missing in ADNI cohort 1.

Supplementary Table S2. Shapiro-Wilk tests for the normality of 16 QBs at the baseline in ADNI cohorts 1, GO/2, and 1/GO/2.

Category	Trait	ADNI cohort 1 ($N = 741$)	ADNI cohort GO/2 ($N = 792$)	ADNI cohort 1/GO/2 ($N = 1,546$)
Cognition	ADAS13	1.36×10^{-9}	1.97×10^{-21}	1.16×10^{-22}
Cognition	CDRSB	7.67×10^{-25}	1.70×10^{-29}	5.27×10^{-37}
Cognition	RAVLT.learning	8.28×10^{-11}	5.96×10^{-9}	6.41×10^{-15}
Cognition	MMSE	2.14×10^{-19}	6.90×10^{-28}	1.07×10^{-32}
Cognition	FAQ	1.68×10^{-30}	1.31×10^{-36}	1.09×10^{-44}
CSF	CSF ABETA	1.94×10^{-16}	4.70×10^{-21}	3.20×10^{-26}
CSF	CSF TAU	4.86×10^{-15}	1.30×10^{-22}	1.55×10^{-26}
CSF	CSF PTAU	7.27×10^{-15}	1.34×10^{-24}	3.71×10^{-28}
PET	FDG PET	1.67×10^{-6}	1.37×10^{-21}	3.58×10^{-22}
PET	Amyloid PET/AV45[a]	-	1.60×10^{-19}	1.60×10^{-19}
MRI	FS WholeBrain	1.01×10^{-2}	1.39×10^{-1}	4.80×10^{-3}
MRI	FS Hippocampus	4.82×10^{-3}	3.95×10^{-3}	1.83×10^{-4}
MRI	FS Entorhinal	2.03×10^{-2}	2.82×10^{-1}	1.60×10^{-2}
MRI	FS Ventricles	8.96×10^{-20}	6.87×10^{-21}	1.16×10^{-28}
MRI	FS MidTemp	4.14×10^{-1}	1.83×10^{-1}	6.96×10^{-1}
MRI	FS Fusiform	6.01×10^{-1}	6.08×10^{-3}	6.02×10^{-3}

[a]The values of Amyloid PET/AV45 at baseline are missing in ADNI cohort 1.

Supplementary Table S3. Longitudinal measures of 16 QBs per visit available in ADNI cohort 1 ($N = 741$).[a]

Category	Trait	bl	m03	m06	m12	m24	m48
Cognition	ADAS13	733 (98.92)	0 (0)	695 (93.79)	653 (88.12)	573 (77.33)	244 (32.93)
Cognition	CDRSB	741 (100.00)	0 (0)	701 (94.60)	656 (88.53)	580 (78.27)	248 (33.47)
Cognition	RAVLT.learning	738 (99.60)	0 (0)	703 (94.87)	657 (88.66)	575 (77.60)	246 (33.20)
Cognition	MMSE	741 (100.00)	0 (0)	705 (95.14)	662 (89.34)	589 (79.49)	248 (33.47)
Cognition	FAQ	738 (99.60)	0 (0)	704 (95.01)	660 (89.07)	582 (78.54)	249 (33.60)
CSF	CSF ABETA	364 (49.12)	0 (0)	1 (0.13)	293 (39.54)	90 (12.15)	65 (8.77)
CSF	CSF TAU	364 (49.12)	0 (0)	1 (0.13)	293 (39.54)	90 (12.15)	65 (8.77)
CSF	CSF PTAU	364 (49.12)	0 (0)	1 (0.13)	293 (39.54)	90 (12.15)	65 (8.77)
PET	FDG PET	366 (49.39)	0 (0)	337 (45.48)	307 (41.43)	265 (35.76)	109 (14.71)
PET	Amyloid PET/AV45	0 (0)	0 (0)	0 (0)	0 (0)	0 (0)	83 (11.20)
MRI	FS WholeBrain	731 (98.65)	0 (0)	668 (90.15)	618 (83.40)	507 (68.42)	199 (26.86)
MRI	FS Hippocampus	606 (81.78)	0 (0)	566 (76.38)	500 (67.48)	398 (53.71)	168 (22.67)
MRI	FS Entorhinal	606 (81.78)	0 (0)	566 (76.38)	500 (67.48)	398 (53.71)	165 (22.27)
MRI	FS Ventricles	728 (98.25)	0 (0)	664 (89.61)	614 (82.86)	502 (67.75)	189 (25.51)
MRI	FS MidTemp	606 (81.78)	0 (0)	566 (76.38)	500 (67.48)	398 (53.71)	165 (22.27)
MRI	FS Fusiform	606 (81.78)	0 (0)	566 (76.38)	500 (67.48)	398 (53.71)	165 (22.27)

bl, baseline visit; m03, month 3 visit; m06, month 6 visit; m12, month 12 visit; m24, month 24 visit; m48, month 48 visit.
[a]Data are described as No. (%).

Supplementary Table S4. Longitudinal measures of 16 QBs per visit available in ADNI cohort GO/2 ($N = 792$).[a]

Category	Trait	bl	m03	m06	m12	m24	m48
Cognition	ADAS13	788 (99.49)	0 (0)	724 (91.41)	642 (81.06)	559 (70.58)	342 (43.18)
Cognition	CDRSB	792 (100.00)	0 (0)	724 (91.41)	639 (80.68)	566 (71.46)	342 (43.18)
Cognition	RAVLT.learning	791 (99.87)	0 (0)	724 (91.41)	640 (80.81)	558 (70.45)	337 (42.55)
Cognition	MMSE	792 (100.00)	0 (0)	729 (92.05)	645 (81.44)	564 (71.21)	342 (43.18)
Cognition	FAQ	790 (99.75)	0 (0)	723 (91.29)	644 (81.31)	570 (71.97)	342 (43.18)
CSF	CSF ABETA	718 (90.66)	0 (0)	1 (0.13)	3 (0.38)	302 (38.13)	98 (12.37)
CSF	CSF TAU	718 (90.66)	0 (0)	1 (0.13)	3 (0.38)	302 (38.13)	97 (12.25)
CSF	CSF PTAU	718 (90.66)	0 (0)	1 (0.13)	3 (0.38)	302 (38.13)	97 (12.25)
PET	FDG PET	783 (98.86)	0 (0)	0 (0)	2 (0.25)	306 (38.64)	3 (0.38)
PET	Amyloid PET/AV45	777 (98.11)	0 (0)	0 (0)	3 (0.38)	479 (60.48)	255 (32.20)
MRI	FS WholeBrain	760 (95.96)	637 (80.43)	573 (72.35)	576 (72.73)	477 (60.23)	212 (26.77)
MRI	FS Hippocampus	722 (91.16)	589 (74.37)	535 (67.55)	564 (71.21)	499 (63.01)	247 (31.19)
MRI	FS Entorhinal	687 (86.74)	562 (70.96)	499 (63.01)	498 (62.88)	402 (50.76)	188 (23.74)
MRI	FS Ventricles	737 (93.06)	609 (76.89)	550 (69.44)	552 (69.70)	444 (56.06)	199 (25.13)
MRI	FS MidTemp	687 (86.74)	562 (70.96)	499 (63.01)	498 (62.88)	402 (50.76)	188 (23.74)
MRI	FS Fusiform	687 (86.74)	562 (70.96)	499 (63.01)	498 (62.88)	402 (50.76)	188 (23.74)

bl, baseline visit; m03, month 3 visit; m06, month 6 visit; m12, month 12 visit; m24, month 24 visit; m48, month 48 visit.

[a]Data are described as No. (%).

Supplementary Table S5. Longitudinal measures of 16 QBs per visit available in ADNI cohort 1/GO/2 ($N = 1{,}546$).[a]

Category	Trait	bl	m03	m06	m12	m24	m48
Cognition	ADAS13	1,534 (99.22)	0 (0)	1,432 (92.63)	1,308 (84.61)	1,144 (74.00)	589 (38.10)
Cognition	CDRSB	1,546 (100.00)	0 (0)	1,438 (93.01)	1,308 (84.61)	1,157 (74.84)	593 (38.36)
Cognition	RAVLT.learning	1,541 (99.68)	0 (0)	1,440 (93.14)	1,310 (84.73)	1,145 (74.06)	586 (37.90)
Cognition	MMSE	1,546 (100.00)	0 (0)	1,447 (93.60)	1,320 (85.38)	1,165 (75.36)	593 (38.36)
Cognition	FAQ	1,541 (99.68)	0 (0)	1,440 (93.14)	1,317 (85.19)	1,163 (75.23)	594 (38.42)
CSF	CSF ABETA	1,089 (70.44)	0 (0)	2 (0.13)	301 (19.47)	392 (25.36)	163 (10.54)
CSF	CSF TAU	1,089 (70.44)	0 (0)	2 (0.13)	301 (19.47)	392 (25.36)	162 (10.48)
CSF	CSF PTAU	1,089 (70.44)	0 (0)	2 (0.13)	301 (19.47)	392 (25.36)	162 (10.48)
PET	FDG PET	1,154 (74.64)	0 (0)	341 (22.06)	313 (20.25)	575 (37.19)	114 (7.37)
PET	Amyloid PET/AV45	777 (50.26)	0 (0)	0 (0)	3 (0.19)	479 (30.98)	340 (21.99)
MRI	FS WholeBrain	1,504 (97.28)	637 (41.20)	1,254 (81.11)	1,206 (78.01)	992 (64.17)	414 (26.78)
MRI	FS Hippocampus	1,338 (86.55)	589 (38.10)	1,113 (71.99)	1,072 (69.34)	902 (58.34)	417 (26.97)
MRI	FS Entorhinal	1,303 (84.28)	562 (36.35)	1,077 (69.66)	1,006 (65.07)	805 (52.07)	355 (22.96)
MRI	FS Ventricles	1,478 (95.60)	609 (39.39)	1,227 (79.37)	1,178 (76.20)	954 (61.71)	391 (25.29)
MRI	FS MidTemp	1,303 (84.28)	562 (36.35)	1,077 (69.66)	1,006 (65.07)	805 (52.07)	355 (22.96)
MRI	FS Fusiform	1,303 (84.28)	562 (36.35)	1,077 (69.66)	1,006 (65.07)	805 (52.07)	355 (22.96)

bl, baseline visit; m03, month 3 visit; m06, month 6 visit; m12, month 12 visit; m24, month 24 visit; m48, month 48 visit.
[a]Data are described as No. (%).

Supplementary Table S6. Point estimates and 95% confidence intervals of regression coefficients β_{f1}, β_{f2}, and β_{m1} in QXcat and QZ_{max} for six statistically significantly associated SNPs found in cross-sectional XWAS.

SNP	Trait	ADNI cohort	β_{f1}		β_{f2}		β_{m1}	
			**Point estimate**	**95% CI**	**Point estimate**	**95% CI**	**Point estimate**	**95% CI**
rs5927116	FS Entorhinal	GO/2	383.656	202.911 ~ 564.401	20.785	-139.401 ~ 180.971	103.977	28.151 ~ 179.804
rs4596772	FS MidTemp	1	-1877.788	-2806.175 ~ -949.400	-259.028	-1006.061 ~ 488.004	-464.309	-790.651 ~ -137.966
rs5929538	FDG PET[a]	1	0.223	-0.200 ~ 0.646	-1.175	-1.616 ~ -0.735	0.103	-0.032 ~ 0.239
rs2213488[b]	FS Hippocampus	GO/2	-331.833	-816.138 ~ 152.471	-326.206	-555.157 ~ -97.255	-91.403	-207.821 ~ 25.016
rs5920524	FDG PET[a]	GO/2	-0.074	-0.346 ~ 0.199	0.465	0.223 ~ 0.707	0.198	0.101 ~ 0.295
rs5945306	FAQ[a]	1/GO/2	-0.639	-0.899 ~ -0.380	-0.053	-0.216 ~ 0.109	-0.004	-0.093 ~ 0.084

CI, confidence interval.

[a]FDG PET and FAQ are transformed using the rank-based inverse normal transformation.

[b]SNP rs2213488 is an overlapping variant in both ADNI cohorts 1 and GO/2. However, it demonstrates the statistical significance only in the analysis of ADNI cohort GO/2 with 792 subjects, while it is not statistically significant in the analysis of ADNI cohort 1/GO/2 with 1,546 subjects ($p_{\mathrm{QMVX_{cat}}} = 6.16 \times 10^{-5}$, $p_{\mathrm{QMVZ_{max}}} = 1.13 \times 10^{-4}$, $p_{\mathrm{QXcat}} = 5.06 \times 10^{-4}$, $p_{\mathrm{QZ_{max}}} = 9.77 \times 10^{-4}$, $p_{\mathrm{T_{chenw}}} = 7.86 \times 10^{-4}$, $p_{\mathrm{T_{plinkw}}} = 3.56 \times 10^{-4}$, and $p_{\mathrm{wM3VNA3.3}} = 9.17 \times 10^{-3}$).

Supplementary Table S7. Point estimates and 95% confidence intervals of regression coefficients δ_{G1}, δ_{G2}, and δ_{G1S} in stage 2 of wM3VNA3.3 for six statistically significantly associated SNPs found in cross-sectional XWAS.

SNP	Trait[a]	ADNI cohort	δ_{G1}		δ_{G2}		δ_{G1S}	
			Point estimate	95% CI	Point estimate	95% CI	Point estimate	95% CI
rs5927116	FS Entorhinal	GO/2	0.015	-0.139 ~ 0.169	-0.089	-0.288 ~ 0.110	0.052	-0.150 ~ 0.253
rs4596772	FS MidTemp	1	0.029	-0.143 ~ 0.202	0.012	-0.211 ~ 0.234	0.010	-0.212 ~ 0.231
rs5929538	FDG PET[a]	1	0.069	-0.194 ~ 0.333	0.067	-0.236 ~ 0.371	0.068	-0.237 ~ 0.372
rs2213488[b]	FS Hippocampus	GO/2	0.300	0.161 ~ 0.439	0.280	0.007 ~ 0.553	-0.341	-0.536 ~ -0.147
rs5920524	FDG PET[a]	GO/2	-0.011	-0.160 ~ 0.139	0.078	-0.095 ~ 0.251	0.118	-0.069 ~ 0.306
rs5945306	FAQ[a]	1/GO/2	0.013	-0.088 ~ 0.114	-0.276	-0.519 ~ -0.034	0.008	-0.139 ~ 0.156

CI, confidence interval.

[a]FDG PET and FAQ are transformed using the rank-based inverse normal transformation.

[b]SNP rs2213488 is an overlapping variant in both ADNI cohorts 1 and GO/2. However, it demonstrates the statistical significance only in the analysis of ADNI cohort GO/2 with 792 subjects, while it is not statistically significant in the analysis of ADNI cohort 1/GO/2 with 1,546 subjects ($p_{\mathrm{QMVX_{cat}}} = 6.16 \times 10^{-5}$, $p_{\mathrm{QMVZ_{max}}} = 1.13 \times 10^{-4}$, $p_{\mathrm{QXcat}} = 5.06 \times 10^{-4}$, $p_{\mathrm{QZ_{max}}} = 9.77 \times 10^{-4}$, $p_{\mathrm{T_{chenw}}} = 7.86 \times 10^{-4}$, $p_{\mathrm{T_{plinkw}}} = 3.56 \times 10^{-4}$, and $p_{\mathrm{wM3VNA3.3}} = 9.17 \times 10^{-3}$).

Supplementary Table S8. Estimates of time×SNP interaction effects in longitudinal XWAS for six SNPs found by cross-sectional XWAS.

SNP	Trait	ADNI cohort	XCI-R			XCI-E		
			Time×SNP	95% CI	*p*-value	Time×SNP	95% CI	*p*-value
rs5927116	FS Entorhinal	GO/2	-5.042	-13.302 ~ 3.218	0.231	-3.124	-13.898 ~ 7.650	0.569
rs4596772	FS MidTemp	1	16.822	-9.825 ~ 43.469	0.215	3.413	-33.608 ~ 40.434	0.856
rs5929538	FDG PET[a]	1	0.002	-0.013 ~ 0.017	0.796	-0.007	-0.029 ~ 0.014	0.492
rs2213488	FS Hippocampus	GO/2	3.167	-11.299 ~ 4.966	0.444	-4.743	-15.909 ~ 6.422	0.404
rs5920524	FDG PET[a]	GO/2	0.010	-0.007 ~ 0.027	0.243	-0.001	-0.024 ~ 0.022	0.926
rs5945306	FAQ[a]	1/GO/2	0.004	-0.009 ~ 0.017	0.577	0.003	-0.016 ~ 0.021	0.792

CI, confidence interval.

[a]FDG PET and FAQ are transformed using the rank-based inverse normal transformation.

Supplementary Table S9. Estimates of SNP main effects in longitudinal XWAS for six SNPs found by cross-sectional XWAS.

SNP	Trait	ADNI cohort	XCI-R			XCI-E		
			SNP	95% CI	p-value	SNP	95% CI	p-value
rs5927116	FS Entorhinal	GO/2	105.116	46.865 ~ 163.367	4.22×10^{-4}	151.225	66.860 ~ 235.590	4.60×10^{-4}
rs4596772	FS MidTemp	1	-531.824	-783.719 ~ -279.929	3.82×10^{-5}	-781.287	-1155.252 ~ -407.322	4.59×10^{-5}
rs5929538	FDG PET[a]	1	-0.022	-0.127 ~ 0.082	6.77×10^{-1}	-0.042	-0.191 ~ 0.107	5.79×10^{-1}
rs2213488	FS Hippocampus	GO/2	-154.842	-244.264 ~ -65.421	7.11×10^{-4}	-269.974	-400.657 ~ -139.292	5.52×10^{-5}
rs5920524	FDG PET[a]	GO/2	0.185	0.101 ~ 0.270	1.99×10^{-5}	0.245	0.131 ~ 0.360	2.80×10^{-5}
rs5945306	FAQ[a]	1/GO/2	-0.044	-0.118 ~ 0.030	2.39×10^{-1}	-0.078	-0.183 ~ 0.027	1.46×10^{-1}

CI, confidence interval.
[a]FDG PET and FAQ are transformed using the rank-based inverse normal transformation.

Supplementary Table S10. Estimates of SNP main effects for nine statistically significantly associated SNPs found in longitudinal XWAS.

SNP	Trait	ADNI cohort	XCI-R			XCI-E		
			SNP	95% CI	*p*-value	SNP	95% CI	*p*-value
rs12157031	FS MidTemp	1/GO/2	78.821	-263.739 ~ 421.381	0.652	179.044	-340.897 ~ 698.984	0.499
rs428303	FS Ventricles[a]	1/GO/2	0.043	-0.019 ~ 0.104	0.172	0.083	-0.001 ~ 0.168	0.053
rs4829868	FS Ventricles[a]	1/GO/2	-0.043	-0.150 ~ 0.064	0.429	-0.005	-0.159 ~ 0.148	0.946
rs5931111	FS Ventricles[a]	1	-0.047	-0.185 ~ 0.091	0.502	-0.002	-0.194 ~ 0.191	0.987
rs5953487	FS Ventricles[a]	1/GO/2	-0.020	-0.083 ~ 0.043	0.531	-0.005	-0.092 ~ 0.082	0.914
rs10284107	FS Ventricles[a]	1/GO/2	0.035	-0.036 ~ 0.107	0.334	0.067	-0.036 ~ 0.170	0.202
rs5955016	FS Ventricles[a]	1/GO/2	0.055	-0.005 ~ 0.116	0.073	0.099	0.016 ~ 0.182	0.020
rs6540385	FS Ventricles[a]	1/GO/2	-0.008	-0.068 ~ 0.052	0.798	0.015	-0.067 ~ 0.096	0.724
rs763320	FS Ventricles[a]	1/GO/2	-0.011	-0.070 ~ 0.049	0.730	0.011	-0.070 ~ 0.093	0.785

CI, confidence interval.

[a]FS Ventricles is transformed using the rank-based inverse normal transformation.

Supplementary Table S11. p-values of all the methods in cross-sectional XWAS for nine SNPs found by longitudinal XWAS.

SNP	Trait	ADNI cohort	$\mathbf{QMVX_{cat}}$	$\mathbf{QMVZ_{max}}$	QXcat	$\mathbf{QZ_{max}}$	$\mathbf{T_{chenw}}$	$\mathbf{T_{plinkw}}$	wM3VNA3.3
rs12157031	FS MidTemp	1/GO/2	6.68×10^{-3}	9.79×10^{-3}	9.25×10^{-3}	1.43×10^{-2}	1.16×10^{-2}	4.32×10^{-1}	8.92×10^{-2}
rs428303	FS Ventricles[a]	1/GO/2	8.09×10^{-2}	9.42×10^{-2}	3.10×10^{-1}	3.75×10^{-1}	2.15×10^{-1}	3.78×10^{-1}	5.06×10^{-2}
rs4829868	FS Ventricles[a]	1/GO/2	9.09×10^{-1}	8.63×10^{-1}	7.94×10^{-1}	6.89×10^{-1}	8.49×10^{-1}	6.73×10^{-1}	7.60×10^{-1}
rs5931111	FS Ventricles[a]	1	9.46×10^{-2}	8.47×10^{-2}	2.01×10^{-1}	1.75×10^{-1}	3.65×10^{-1}	3.03×10^{-1}	9.48×10^{-2}
rs5953487	FS Ventricles[a]	1/GO/2	2.48×10^{-2}	1.55×10^{-2}	1.44×10^{-1}	8.31×10^{-2}	3.81×10^{-1}	2.29×10^{-1}	2.61×10^{-2}
rs10284107	FS Ventricles[a]	1/GO/2	8.34×10^{-1}	8.25×10^{-1}	1.00	9.74×10^{-1}	6.82×10^{-1}	8.57×10^{-1}	4.82×10^{-1}
rs5955016	FS Ventricles[a]	1/GO/2	3.71×10^{-1}	2.74×10^{-1}	3.50×10^{-1}	2.28×10^{-1}	6.06×10^{-1}	4.12×10^{-1}	3.37×10^{-1}
rs6540385	FS Ventricles[a]	1/GO/2	2.25×10^{-1}	1.83×10^{-1}	7.23×10^{-1}	5.48×10^{-1}	8.04×10^{-1}	8.29×10^{-1}	8.12×10^{-2}
rs763320	FS Ventricles[a]	1/GO/2	2.86×10^{-1}	2.37×10^{-1}	5.21×10^{-1}	4.02×10^{-1}	6.58×10^{-1}	7.29×10^{-1}	1.56×10^{-1}

[a]FS Ventricles is transformed using the rank-based inverse normal transformation.

Supplementary Table S12. p-values of cross-sectional XWAS only based on non-Hispanic White subjects for six SNPs, which were identified to be statistically significant in cross-sectional XWAS based on the cross-ethnic sample.[a]

SNP	Trait	ADNI cohort	$QMVX_{cat}$	$QMVZ_{max}$	QXcat	QZ_{max}	T_{chenw}	T_{plinkw}	wM3VNA3.3
rs5927116	FS Entorhinal	GO/2	6.34×10^{-6}	9.62×10^{-6}	$\mathbf{6.82 \times 10^{-7}}$	$\mathbf{1.07 \times 10^{-6}}$	$\mathbf{2.00 \times 10^{-6}}$	1.53×10^{-5}	5.91×10^{-1}
rs4596772	FS MidTemp	1	1.30×10^{-4}	6.83×10^{-5}	1.25×10^{-5}	6.21×10^{-6}	5.58×10^{-5}	1.14×10^{-4}	8.35×10^{-1}
rs5929538	FDG PET[b]	1	8.99×10^{-4}	1.67×10^{-1}	2.68×10^{-4}	1.22×10^{-1}	6.67×10^{-6}	1.43×10^{-3}	3.25×10^{-1}
rs2213488	FS Hippocampus	GO/2	3.26×10^{-5}	2.41×10^{-5}	4.76×10^{-4}	3.43×10^{-4}	9.89×10^{-4}	3.18×10^{-4}	4.91×10^{-3}
rs5920524	FDG PET[b]	GO/2	$\mathbf{1.20 \times 10^{-6}}$	2.82×10^{-5}	$\mathbf{1.78 \times 10^{-7}}$	5.15×10^{-6}	$\mathbf{1.27 \times 10^{-7}}$	1.49×10^{-5}	3.87×10^{-1}
rs5945306	FAQ[b]	1/GO/2	$\mathbf{2.47 \times 10^{-6}}$	2.53×10^{-4}	$\mathbf{4.07 \times 10^{-7}}$	5.92×10^{-5}	$\mathbf{4.34 \times 10^{-7}}$	7.05×10^{-5}	3.64×10^{-1}

[a]The p-values less than the significance level of 2.54×10^{-6} are highlighted in bold.
[b]FDG PET and FAQ are transformed using the rank-based inverse normal transformation.

Supplementary Table S13. Estimates of time×SNP interaction effects in longitudinal XWAS only based on non-Hispanic White subjects for nine SNPs, which were identified to be statistically significant in longitudinal XWAS based on the cross-ethnic sample.

SNP	Trait	ADNI cohort	XCI-R			XCI-E		
			Time×SNP	95% CI	p-value[b]	Time×SNP	95% CI	p-value[b]
rs12157031	FS MidTemp	1/GO/2	57.030	32.408 ~ 81.652	5.92×10^{-6}	86.772	46.152 ~ 127.393	5.11×10^{-5}
rs428303	FS Ventricles[a]	1/GO/2	-0.005	-0.009 ~ -0.002	2.80×10^{-3}	-0.010	-0.014 ~ -0.005	2.43×10^{-5}
rs4829868	FS Ventricles[a]	1/GO/2	-0.009	-0.015 ~ -0.003	1.69×10^{-3}	-0.016	-0.023 ~ -0.009	3.48×10^{-5}
rs5931111	FS Ventricles[a]	1	-0.010	-0.017 ~ -0.002	1.84×10^{-2}	-0.017	-0.028 ~ -0.006	3.53×10^{-3}
rs5953487	FS Ventricles[a]	1/GO/2	-0.004	-0.007 ~ -0.001	1.99×10^{-2}	-0.009	-0.014 ~ -0.005	6.08×10^{-5}
rs10284107	FS Ventricles[a]	1/GO/2	-0.007	-0.010 ~ -0.003	4.82×10^{-4}	-0.012	-0.017 ~ -0.007	2.63×10^{-6}
rs5955016	FS Ventricles[a]	1/GO/2	-0.003	-0.007 ~ -0.000	4.27×10^{-2}	-0.008	-0.013 ~ -0.004	3.06×10^{-4}
rs6540385	FS Ventricles[a]	1/GO/2	-0.007	-0.010 ~ -0.004	1.60×10^{-5}	-0.012	-0.016 ~ -0.007	$\mathbf{1.76 \times 10^{-7}}$
rs763320	FS Ventricles[a]	1/GO/2	-0.007	-0.010 ~ -0.004	1.79×10^{-5}	-0.012	-0.016 ~ -0.007	$\mathbf{1.36 \times 10^{-7}}$

CI, confidence interval.

[a]FS Ventricles is transformed using the rank-based inverse normal transformation.

[b]The p-values less than the significance level of 2.54×10^{-6} are highlighted in bold.

Supplementary Table S14. Estimates of SNP main effects in longitudinal XWAS only based on non-Hispanic White subjects for nine SNPs, which were identified to be statistically significant in longitudinal XWAS based on the cross-ethnic sample.

SNP	Trait	ADNI cohort	XCI-R			XCI-E		
			SNP	95% CI	*p*-value	SNP	95% CI	*p*-value
rs12157031	FS MidTemp	1/GO/2	132.294	-242.833 ~ 507.420	0.489	238.967	-345.803 ~ 823.737	0.423
rs428303	FS Ventricles[a]	1/GO/2	0.044	-0.021 ~ 0.110	0.185	0.093	-0.000 ~ 0.186	0.051
rs4829868	FS Ventricles[a]	1/GO/2	-0.070	-0.188 ~ 0.048	0.244	-0.041	-0.215 ~ 0.132	0.640
rs5931111	FS Ventricles[a]	1	-0.126	-0.293 ~ 0.040	0.138	-0.104	-0.349 ~ 0.141	0.407
rs5953487	FS Ventricles[a]	1/GO/2	-0.022	-0.089 ~ 0.046	0.534	-0.001	-0.097~ 0.096	0.987
rs10284107	FS Ventricles[a]	1/GO/2	0.013	-0.064 ~ 0.090	0.734	0.039	-0.072 ~ 0.150	0.490
rs5955016	FS Ventricles[a]	1/GO/2	0.030	-0.034 ~ 0.094	0.364	0.064	-0.026 ~ 0.154	0.163
rs6540385	FS Ventricles[a]	1/GO/2	0.002	-0.061 ~ 0.065	0.947	0.027	-0.060 ~ 0.114	0.546
rs763320	FS Ventricles[a]	1/GO/2	0.001	-0.063 ~ 0.064	0.987	0.024	-0.063 ~ 0.112	0.584

CI, confidence interval.

[a]FS Ventricles is transformed using the rank-based inverse normal transformation.

Supplementary References

Chen, B., Craiu, R.V., Strug, L.J., and Sun, L. (2021). The X factor: a robust and powerful approach to X-chromosome-inclusive whole-genome association studies. *Genet. Epidemiol.* 45**,** 694-709. doi: 10.1002/gepi.22422

Chen, Z., Ng, H.K.T., Li, J., Liu, Q., and Huang, H. (2017). Detecting associated single-nucleotide polymorphisms on the X chromosome in case control genome-wide association studies. *Stat. Methods Med. Res.* 26**,** 567-582. doi: 10.1177/0962280214551815

Deng, W.Q., Mao, S., Kalnapenkis, A., Esko, T., Mägi, R., Paré, G., et al. (2019). Analytical strategies to include the X-chromosome in variance heterogeneity analyses: evidence for trait-specific polygenic variance structure. *Genet. Epidemiol.* 43**,** 815-830. doi: 10.1002/gepi.22247

Fisher, B., Tucson, A., Costich, E.R., Ganz, M., and Stanford, J.W. (1967). Questions & Answers. *J. Am. Dent. Assoc.* 75**,** 799. doi: 10.14219/jada.archive.1967.0319

Özbek, U., Lin, H.M., Lin, Y., Weeks, D.E., Chen, W., Shaffer, J.R., et al. (2018). Statistics for X-chromosome associations. *Genet. Epidemiol.* 42**,** 539-550. doi: 10.1002/gepi.22132

Wang, H., Zhang, F., Zeng, J., Wu, Y., Kemper, K.E., Xue, A., et al. (2019a). Genotype-by-environment interactions inferred from genetic effects on phenotypic variability in the UK Biobank. *Sci. Adv.* 5**,** eaaw3538. doi: 10.1126/sciadv.aaw3538

Wang, J., Yu, R., and Shete, S. (2014). X-chromosome genetic association test accounting for X-inactivation, skewed X-inactivation, and escape from X-inactivation. *Genet. Epidemiol.* 38**,** 483-493. doi: 10.1002/gepi.21814

Wang, P., Xu, S.Q., Wang, B.Q., Fung, W.K., and Zhou, J.Y. (2019b). A robust and powerful test for case-control genetic association study on X chromosome. *Stat. Methods Med. Res.* 28**,** 3260-3272. doi: 10.1177/0962280218799532

Yang, Z.-Y., Liu, W., Yuan, Y.-X., Kong, Y.-F., Zhao, P.-Z., Fung, W.K., et al. (2022). Robust association tests for quantitative traits on the X chromosome. *Heredity* 129**,** 244-256. doi: 10.1038/s41437-022-00560-y

Yu, W.-Y., Zhang, Y., Li, M.-K., Yang, Z.-Y., Fung, W.K., Zhao, P.-Z., et al. (2022). BEXCIS: Bayesian methods for estimating the degree of the skewness of X chromosome inactivation. *BMC Bioinformatics* 23**,** 193. doi: 10.1186/s12859-022-04721-y
